# Dynamic Load Model Systems of Tendon Inflammation and Mechanobiology

**DOI:** 10.3389/fbioe.2022.896336

**Published:** 2022-07-15

**Authors:** Lindsay G. Benage, James D. Sweeney, Morgan B. Giers, Ravi Balasubramanian

**Affiliations:** ^1^ School of Chemical, Biological and Environmental Engineering, Oregon State University, Corvallis, OR, United States; ^2^ School of Mechanical, Industrial and Manufacturing Engineering, Oregon State University, Corvallis, OR, United States

**Keywords:** tendinopathy, tendon, inflammation, mechanotransduction, tendon pathology, extracellular matix, dynamic loading

## Abstract

Dynamic loading is a shared feature of tendon tissue homeostasis and pathology. Tendon cells have the inherent ability to sense mechanical loads that initiate molecular-level mechanotransduction pathways. While mature tendons require physiological mechanical loading in order to maintain and fine tune their extracellular matrix architecture, pathological loading initiates an inflammatory-mediated tissue repair pathway that may ultimately result in extracellular matrix dysregulation and tendon degeneration. The exact loading and inflammatory mechanisms involved in tendon healing and pathology is unclear although a precise understanding is imperative to improving therapeutic outcomes of tendon pathologies. Thus, various model systems have been designed to help elucidate the underlying mechanisms of tendon mechanobiology *via* mimicry of the *in vivo* tendon architecture and biomechanics. Recent development of model systems has focused on identifying mechanoresponses to various mechanical loading platforms. Less effort has been placed on identifying inflammatory pathways involved in tendon pathology etiology, though inflammation has been implicated in the onset of such chronic injuries. The focus of this work is to highlight the latest discoveries in tendon mechanobiology platforms and specifically identify the gaps for future work. An interdisciplinary approach is necessary to reveal the complex molecular interplay that leads to tendon pathologies and will ultimately identify potential regenerative therapeutic targets.

## 1 Introduction

Tendon acute injuries and chronic dysfunction (tendinopathy) are a common issue among athletic and occupational settings. Clinical symptoms of tendinopathy include pain, weakness, and the inability to perform activities of daily life. Risk factors for tendinopathy include age, obesity, activity level, and anatomical asymmetries (Riley, 2005; Lorimer and Hume, 2014, 2016; Thampatty and Wang, 2018). In 2015, musculoskeletal disorders accounted for more than half of the disabling health conditions reported by adults in the US (Weinstein et al., 2014), and tendon and ligament injuries have been estimated to account for 20–30% of all musculoskeletal disorders (Fleming et al., 2005). Despite its prevalence, the etiology of tendon pathologies is not well understood. Consequently, treatment options often do not resolve the issue (Riley, 2005).

**FIGURE 1:**
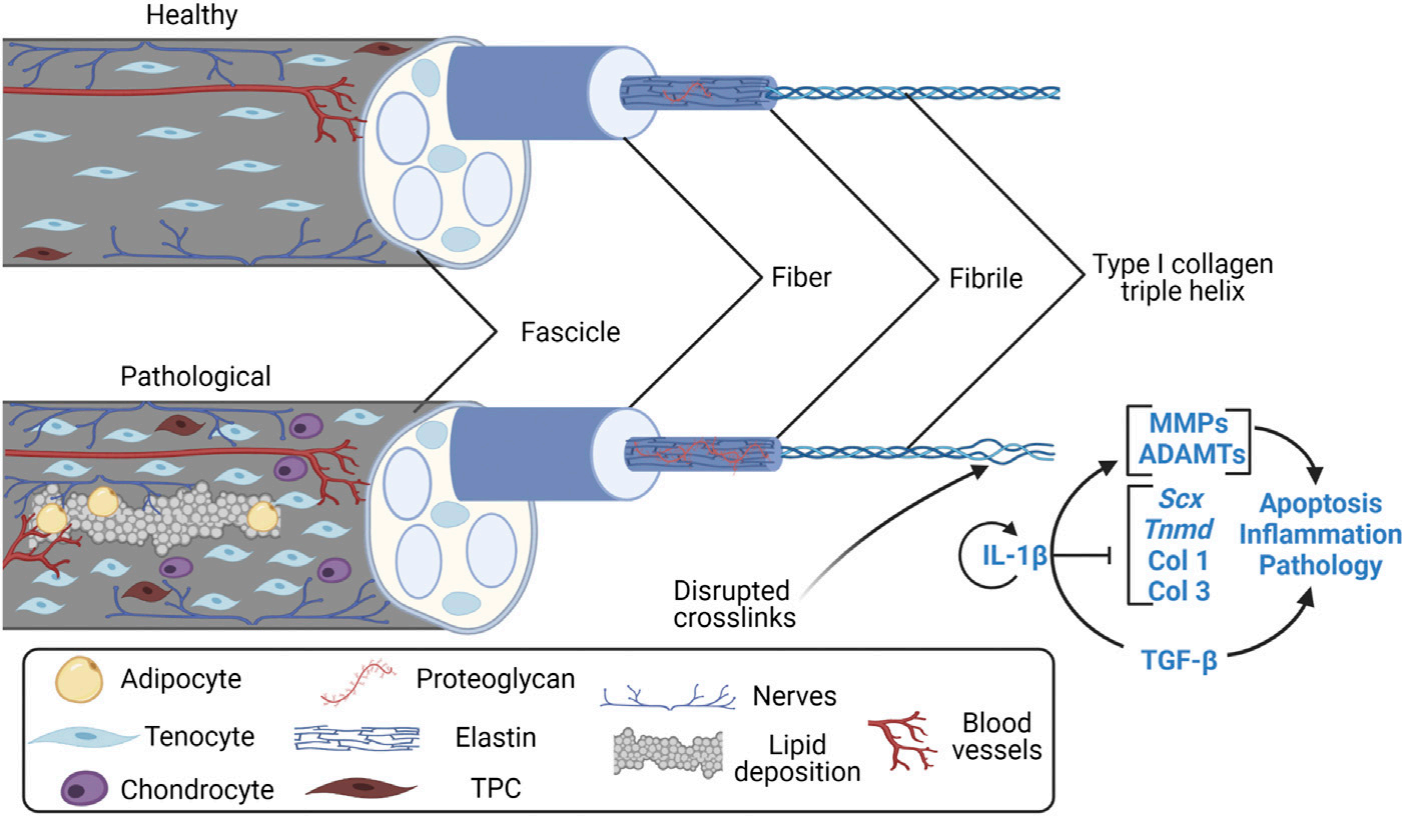
Features of the healthy and pathological tendon fascicle. Created with Biorender.com. Features of the clinically pathological tendon fascicle (a hierarchical subunit of whole tendon) include lipid deposition, angiogenesis, nerve ingrowth, adipocyte and chondrocyte-like TPC differentiation (Kannus and Józsa, 1991; Józsa and Kannus, 1997; Zhang and Wang, 2010, 2014; Agarwal et al., 2017), increases in proteoglycan content (Fu, Chan and Rolf, 2007; Samiric et al., 2009; Attia et al., 2014), collagen type III RNA upregulation/deposition (Liu et al., 1995; Samiric et al., 2009), and irregularities in collagen alignment/crosslinking at previous microscopic to macroscopic rupture sites (Kannus and Józsa, 1991; Józsa and Kannus, 1997; Järvinen et al., 2004). Overall, the pathological tendon is mechanically weaker than the healthy tendon. TPC, tendon progenitor cell; Scx, Scleraxis; Tnmd, Tenomodulin; Col 1, collagen type I; Col 3, collagen type III; MMP, matrix metalloproteinases; ADAMT, disintegrin and metalloproteinase with thrombospondin motifs; IL-1β, interleukin-1β; TGF-β, transforming growth factor β.

Tendinopathy is defined by a failure to heal following tissue trauma that leads to a progressively degenerative imbalance in extracellular matrix (ECM) turnover (Burk, 2019). However, the exact mechanisms for the imbalance are not yet well-characterized. Repetitive low-level dynamic loading or underloading of tissues is implicated in tendinopathy etiology though dynamic loading is also necessary for proper maturation and matrix homeostasis. Thus, the boundaries between underloading, homeostatic loading, and overloading may vary across anatomical location (Johnson et al., 1994; Itoi et al., 1995; Maganaris and Paul, 1999), and within species (Goodship et al., 1994; Maganaris and Paul, 1999; Matson et al., 2012; Zitnay and Weiss, 2018), due to variations in mechanical properties. Tendon injuries may be acute and characterized by inflammation, called “tendonitis”, or they are chronic and characterized by a degenerative ECM, called “tendinosis” (Lipman et al., 2018). Both conditions are incorporated into the more generic term “tendinopathy” that highlights tendon pain, dysfunction, and often an inability to specifically diagnose the disorder. Tendinopathy has been adopted in part due to histological findings that suggest noteworthy absence of inflammatory cells in the later stages of the pathological tendon (Riley, 2005; Legerlotz et al., 2013). Instead, histological characteristics most notably include lipid deposition, increased vascularity, increased proteoglycan content, and calcification (Figure 1) (Kannus and Józsa, 1991). However, recent findings suggest low-levels of inflammation persist throughout the course of tendinopathy (Chisari et al., 2019; Arvind and Huang, 2021). Overall, pathological changes in the tendon, including localized regions of decreased collagen fibre thickness, decreased crimp angle, and heterogeneous crimping, have been associated with tendon rupture (Kannus and Józsa, 1991; Järvinen et al., 2004). Conclusively, inflammation may contribute to the onset of tendinopathies, but its temporal role after initiation needs to be characterized.

It is essential to identify the molecular and mechanical mechanisms involved in tendinopathy etiology in order to improve therapeutic outcomes (Screen et al., 2015). Exploration of these mechanisms can be carried out with *ex vivo* and *in vitro* models. The core focus of this review is recent advancements in the field of tendon mechanobiology using *in vitro* and *ex vivo* platforms and includes several *in vivo* models of tendinopathy, which were selected based on the described methods below. Also included is primary background on tendon physiology and mechanical properties, which is essential to evaluate these model systems. Future directions for this work are emphasized in the gaps in current understanding and among model system platforms.

## 2 Methods

Web of Science and PubMed library searches were conducted using key words or MeSH terms such as “Model” AND “Tendinopathy” OR “Tendinosis” OR “Tendonitis” AND “Tendons” AND “Inflammation”. For the section on model systems of tendinopathy, greater focus was placed on the most recent findings in the years 2015–2022. In this year range, all studies relating to dynamic or cyclical loading-induced effects on tendon modulation and inflammation of *in vitro* and *ex vivo* systems were included. To highlight the limited research in this field, total publications relating to “tendinopathy models” is about 15-fold lower than cartilage “osteoarthritis models” for the years 2011–2021 in PubMed databases (Supplementary Figure S1). Cartilage is also a hypovascular tissue that shares similar degenerative features to the tendon, yet the role of mechanical loading and inflammation is more heavily researched in osteoarthritis etiology (Robinson et al., 2016; Chisari et al., 2019). Key words and MeSH terms of “Tendinopathy” AND “Inflammation” AND “Model” versus “Osteoarthritis” AND “Inflammation” AND “Model” were used for this comparison of published literature on these topics.

## 3 Tendon Physiology

Tendon is a densely collagenous, hypocellular tissue, where ECM turnover is a key component of tendon homeostasis and its ability to sustain loading (Nabeshima et al., 1996; Gracey et al., 2020). During embryonic development, tendon tissue shifts from a hypercellular environment dominated by cell-cell interactions, to predominantly cell-matrix interactions within a hypocellular environment (Cosgrove et al., 2016). Tenocytes are the predominant cell-type in the mature tendon, making up 90–95% of cells yet only 5% of tissue volume (Kannus, 2000; Cosgrove et al., 2016; Wu et al., 2018). They are fully differentiated and are primarily responsible for tissue homeostasis *via* ECM remodeling (Burk, 2019). Meanwhile, the immature tenocytes, or tenoblasts, and tendon progenitor cells (TPCs), make up a large majority of the other cells within the tendon and are necessary for tenocyte proliferation and differentiation, respectively.

Overall, tendon cells are mechanosensing. They are sensitive to various load types (Mendias, Gumucio and Lynch, 2012; Zitnay and Weiss, 2018), frequency (Lavagnino et al., 2003; Kubo et al., 2020), and magnitudes (Screen et al., 2005; Mendias et al., 2012). Neonatal developmental structural and compositional changes of the ECM are associated with increases in load-bearing (Ansorge et al., 2011; Theodossiou et al., 2021). Collagen bundles transfer primarily uniaxial tensile forces to tenocytes while the greater collagen and ECM architecture transfers minor shear and compressive forces during loading (Khan and Scott, 2009). Once a loading threshold has been reached, the cells respond accordingly *via* a complex interplay of cell signaling with possible activation of inflammatory pathways (Gracey et al., 2020). Ultimately, this results in an anabolic, catabolic, or balanced activity in the extracellular matrix.

### 3.1 Extracellular Matrix Composition

The main load-bearing component of the tendon ECM is type I collagen (Col1), which composes 80–90% of tendon dry weight (see Supplementary Figure S2) (Kannus, 2000; Wang et al., 2012). Collagen type I is a heterotrimeric molecule composed of two α1 chains and one α2 chain forming a triple helix. Many collagen types (V, IX, X, XI, and XII) are found in smaller amounts within the tendon, though they still provide important functions. For example, collagen fibril diameter is partially regulated by collagen type V (Birk and Mayne, 1997; Wang et al., 2012). Other components involved in ECM modulation include type III collagen (Col3) which is important for quick cross-linking and stability of injury sites (Liu et al., 1995), integrin transmembrane proteins involved in sensing loads, and proteoglycans (decorin, aggrecan, etc., make up 1–5% of tendon dry weight) that hydrate the tendon and contribute to fibrillar slippage and resisting compression, respectively (Fessel and Snedeker, 2009; Wang et al., 2012; Screen et al., 2015). Matrix metalloproteinases (MMPs, mainly 1, 3, and 12) and ADAMTS-1, -4, -5, -8, -9, -15, and -20 (disintegrin and metalloproteinase with thrombospondin motifs, or aggrecanases) enzymatically breakdown various extracellular matrix components (Buono et al., 2013). Glycoproteins such as fibronectin, elastin (1–2% of tendon dry weight) (Kannus, 2000), and tenascin-C contribute to tendon repair and the elastic properties of the tendon (Wang et al., 2012). Intermolecular enzymatic cross-linking is an interaction mainly between thick Col1 fibrils, thinner Col3 fibrils, elastin, tenascin-C (Midwood et al., 2016), and proteoglycan components that occurs during maturation and adaptation to mechanical loads (Eyre et al., 1984; Kubow et al., 2015; Zitnay and Weiss, 2018). Consequently, the architectural arrangement of cross-linked components influences the load-bearing ECM-derived functional properties (Kjaer, 2004).

### 3.2 Tendon Signaling

**FIGURE 2:**
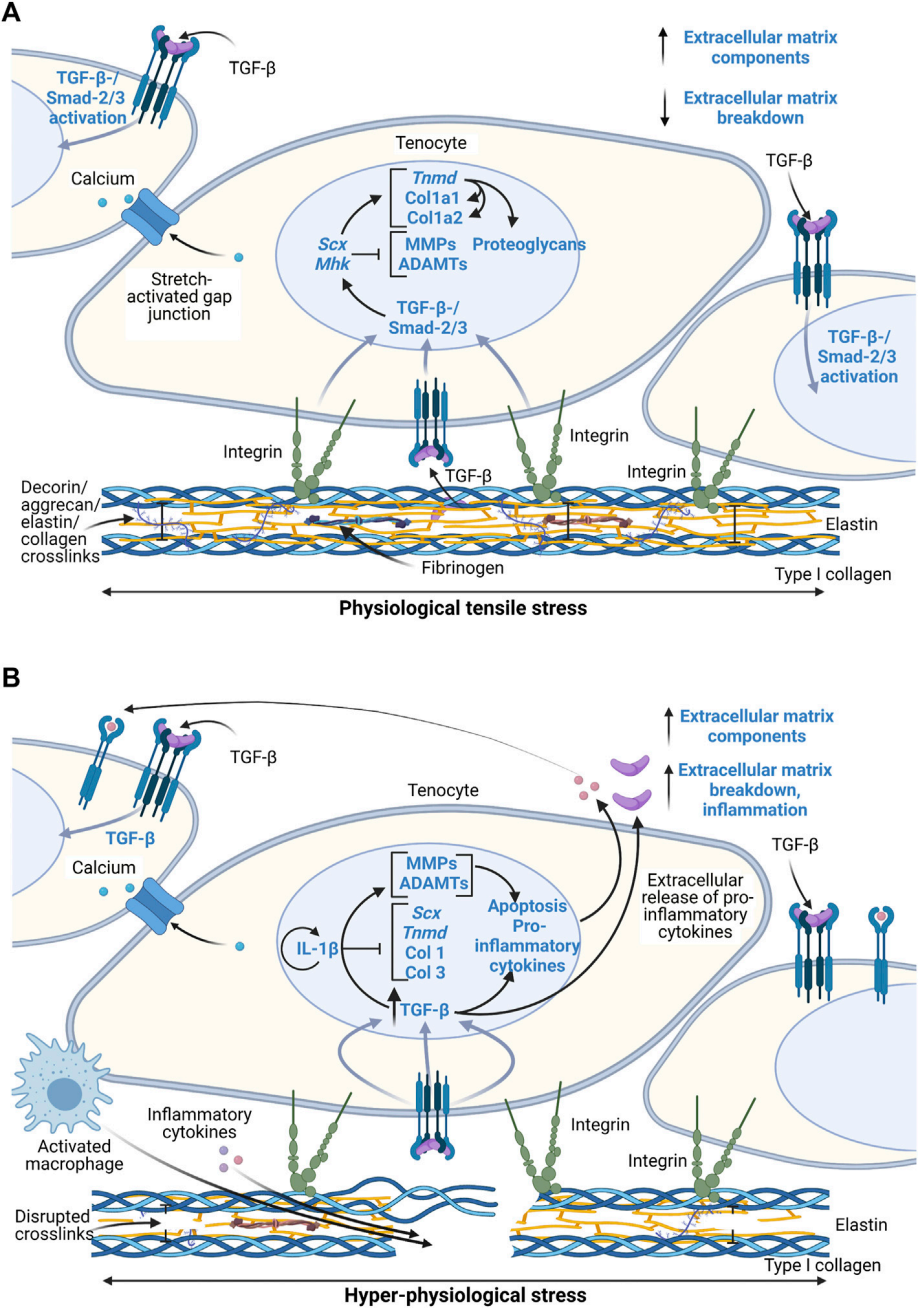
Schematic of proposed tenocyte molecular response to physiological and hyper-physiological mechanical loading thresholds. Created with Biorender.com. **(A)** Molecular cascade following physiological loading resulting in both integrin-mediated pathways and postulated stretch-activated ion channel pathways to induce a TGF-β-/Smad-2/3 activation and thus transcription of various extracellular matrix related genes and regulatory enzymes. **(B)** Hyper-physiological loading leads to macro-scale ruptures that induce inflammatory cascades through greater activation of TGF-β pathways and IL-1β that lead to paracrine signaling and ultimately matrix degradation and inflammatory cell localization at the injury site. Scx, Scleraxis; Tnmd, Tenomodulin; Col 1, collagen type I; Col 3, collagen type III; MMP, matrix metalloproteinases; ADAMT, disintegrin and metalloproteinase with thrombospondin motifs; IL-1β, interleukin-1β; TGF-β, transforming growth factor β.

Transforming growth factor beta (TGF-β) is highly correlated to mechanical stress intensity and thus the tendon’s mechanoresponse *via* the Smad2/3 pathway (Figure 2A) (Dahlgren et alk., 2005; Maeda et al., 2011; Munger and Sheppard, 2011; Mendias et al., 2012). Downstream of the Smad2/3 pathway, Scleraxis (*Scx*), Mohawk (*Mhk*), and Tenomodulin (*Tnmd*) are tenocyte-specific markers that regulate proteoglycan production and collagen synthesis (Berthet et al., 2013; Subramanian et al., 2017). In developing mouse tendons, *Tnmd* expression is downstream of *Scx*, though they both likely play a role in Col1 transcription and adaptation to mechanical loading as well as tenocyte proliferation (Léjard et al., 2007). These markers have been identified as significant factors involved in tendon homeostasis and degeneration. They are heavily studied in model systems though exact mechanisms have not been clearly identified in all loading scenarios.

Current knowledge of the interaction between integrin proteins in cells and the ECM during loading is limited. It is unclear whether or not the cells respond to loads primarily *via* an integrin-mediated pathway or *via* stretch-activated ion channels that are caused by actual deformation of the cytoskeleton and nucleus in the cell. Decoupling of the mechanosensing process during maturation can be achieved using *in vitro* models of stem cell differentiation and ECM maturation in response to mechanical loads (Cosgrove et al., 2016). In other words, defining the cell-matrix interactions versus cell-cell interactions is necessary using simplified models.

### 3.3 Inflammation and Matrix Modulation in Tendinopathy

**FIGURE 3:**
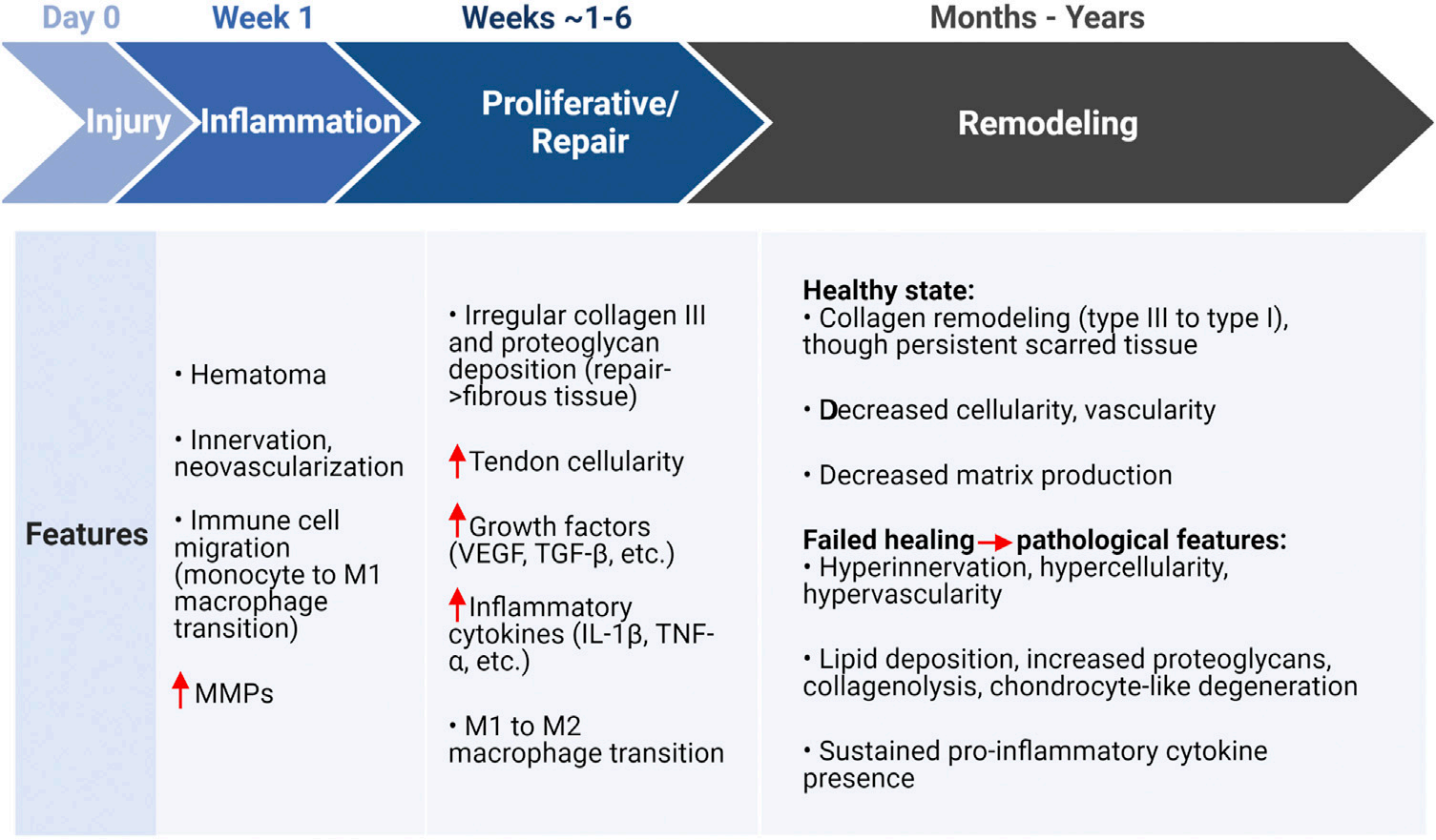
Tendon healing estimated timeline and features associated with a healthy or pathological state following injury. Created with Biorender.com. Initial injury due to overloading induces an inflammatory state that is characterized by matrix degradation, cell apoptosis, and immune cell localization (monocytes) that transition to a pro-inflammatory (M1) macrophage for removal of cell and ECM debris. The proliferative or repair phase (estimated 1–6 weeks) is characterized by injury site repair via increased cellularity, growth factors, inflammatory cytokines, and eventually the inflammation resolving M2 macrophage transition. The final remodeling stage can take months to years, where pathological features may persist with sustained inflammatory cytokines (though not immune cells) and degeneration of the ECM. In either the healthy or pathological case, a persistent scar-like tissue remains present (Fu et al., 2010; Lipman et al., 2018; Alim et al., 2020; Gracey et al., 2020). MMPs, matrix metalloproteinases; VEGF, vascular endothelial growth factor; TGF-β, transforming growth factor β; IL-1β, interleukin-1β; TNF-α, tumor necrosis factor α.

Inflammation is vital to healing of acute injuries, yet the tendon has low regeneration potential once past early post-natal stages (Ansorge et al., 2012; Howell et al., 2017; Stauber et al., 2020). Thus, it is necessary for inflammatory pathways to be resolved to limit tissue damage and prevent continual degradation. The timeline for tendon healing is estimated (Figure 3) where pro-inflammatory signaling resolves between about week 1 and 6 post-injury, yet the underlying mechanisms for resolving inflammation (or not resolving inflammation) is unclear. Endogenous TPCs are capable of regenerating tenocytes, but this ability becomes limited due to aging and their susceptibility to injury (Xu and Liu, 2018). Furthermore, TPCs are implicated in fatty infiltration, fibrosis, and calcification in tendinopathy through TPC differentiation into adipocytes and chondrocytes (Figure 1), signaled by high levels of the pro-inflammatory cytokine prostaglandin E2 (PGE2) (Zhang and Wang, 2010, 2014; Agarwal et al., 2017).

Mechanical micro-ruptures and metabolic byproducts during repetitive or hyper-physiological loading stresses the tendon cells. Ultimately, this triggers an inflammatory cascade, leading to ECM degradation and further pathological characteristics (Figure 2B). Other factors highly involved in these pathways include pro-inflammatory cytokines interleukin-1 (IL-1) and tumor necrosis factor (TNF)-α (John et al., 2010), glutamate, vascular endothelial growth factor (VEGF), and substance P (Abate et al., 2009; Schulze-Tanzil et al., 2011). These signals activate angiogenesis and nerve ingrowth, leading to pain. In addition, they contribute to fibrosis formation due to macrophage signaling and localization and thus fibroblast activation (Dakin et al., 2012, 2017). Inflammatory pathways lead to ECM degradation and mechanical weakening of ECM *via* MMP upregulation and downregulation of tissue inhibitors of MMPs (Abate et al., 2009; Buono et al., 2013). Prolonged presence of macrophages further contributes to degradation of the ECM as they also secrete MMPs. Overall, micro-ruptures may also contribute to the onset of tendinopathy through the inability to properly heal or resolve inflammation before reoccurrence.

## 4 Tendon Mechanical Properties

**FIGURE 4:**
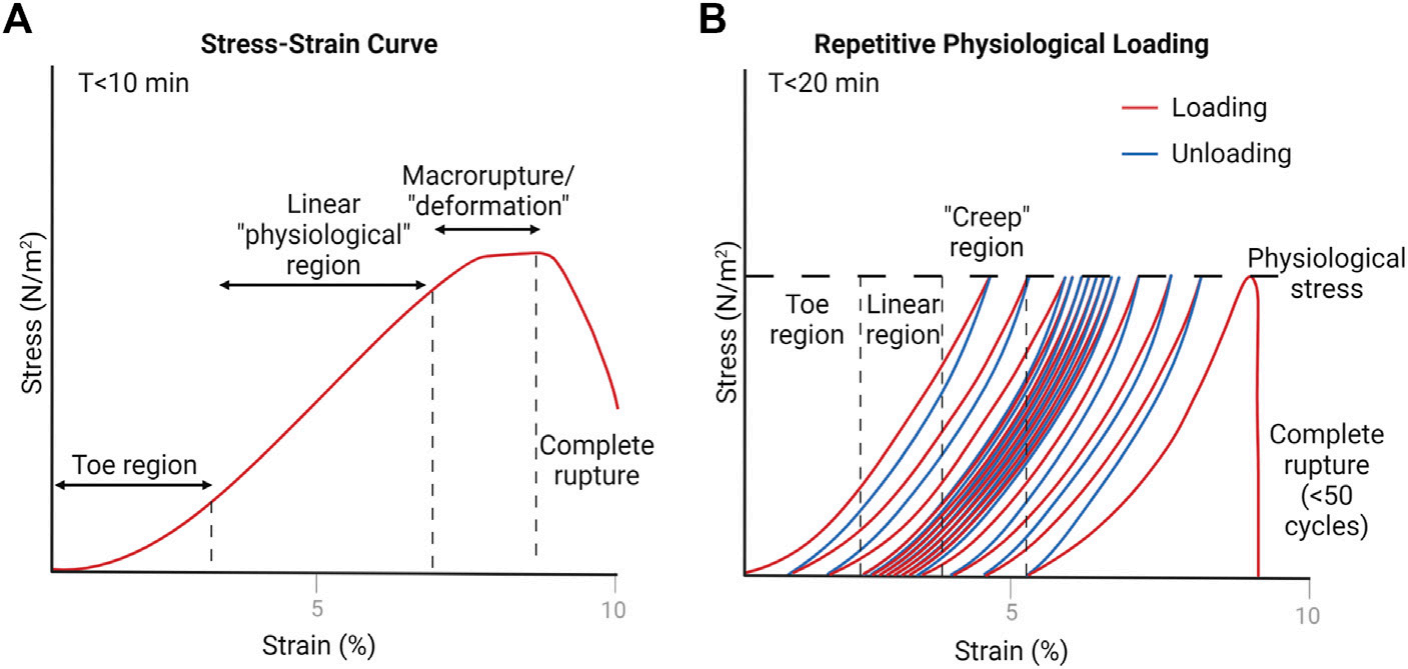
Representative estimations of tendon loading schemes. Created with Biorender.com. **(A)** Pull-to-failure test results in a stress-strain curve when normalized to tendon cross-sectional area (Wang, 2006; Wang et al., 2012). The toe region is due to natural collagen fiber crimping, the linear region is equated to physiological stress values, and the deformation region is where macro-scale ruptures start to occur (Louis-Ugbo et al., 2004). **(B)** Fatigue test indicates similar regions that occur due to repetitive physiological loading, where “creep” occurs due to macro-scale ruptures eventually leading to complete rupture of the tendon after a number of cycles (usually less than 50) (Thornton et al., 2002).

Tendon’s passive, viscoelastic properties are dependent on species, age, gender, and anatomical location (Johnson et al., 1994; Itoi et al., 1995; Maganaris and Paul, 1999; Louis-Ugbo et al., 2004; Burgio et al., 2022). They have unique and identifiable material properties, including Young’s Modulus and ultimate failure force. In tendon mechanobiology studies, these properties are often quantified in conjunction with other physiological and structural changes. The two most common mechanical tests include a tensile pull-to-failure, or ultimate stress test (Figure 4A) and a fatigue, or creep test (Figure 4B).

In ultimate stress tests, the tendon is pulled along the longitudinal axis at a specified rate, and the stress or force values are often measured alongside tissue strain or deformation to form a stress-strain curve. From this curve, Young’s Modulus is determined by the slope of the linear region—representing tendon stiffness. Loading of the *in vivo* tendon in the linear range is considered physiological loading (generally 2–6% strain) (Wang and Chen, 2018). Various types of loading, even in the physiological range, can cause micro-tearing within the tendon. After the linear range, the tendon starts to experience plastic deformation, or macro-ruptures, and ultimately leads to complete rupture of the tendon. At this point, the ultimate stress value is defined.

Measured values of Young’s Modulus and ultimate stress will vary drastically across species, the specific tendon, and the loading rate used to perform the tensile test (Burgio et al., 2022). For a loading rate of 0.1 mm/s, rat Achilles tendon measurements for Young’s Modulus and ultimate stress range between 179 ± 36 MPa and 45 ± 10 MPa, respectively (Eliasson et al., 2007). For a loading rate of 1 mm/s, however, the Young’s Modulus (405 ± 115 MPa) was much greater for the rat Achilles tendon as well as its ultimate stress values (51.6 ± 10.8 MPa) (Legerlotz et al., 2007). Furthermore, the human Achilles tendon Young’s Modulus of 816 ± 218 MPa and ultimate stress of 7.5 ± 1.1 MPa, measured at a rate of 1 mm/s, vary greatly from the rat (Wren et al., 2001). Burgio et al., 2022, covers the mechanical properties of various tendons in-depth (Burgio et al., 2022).

Fatigue tests provide insight on the tendon’s ability to repeatedly sustain loads, such as in running or walking. The setup is similar to ultimate stress tests, except that the tendon is uniaxially pulled to a specified force under a specific loading rate, then released, and repeated at some frequency. Because of the viscoelastic nature of the tendon, repetitive loading leads to an increase in deformation or strain (“creep”) at the specified force. After a number of cycles (less than 50), the tendon suffers macro-ruptures and eventually ruptures completely (Wang and Chen, 2018).

Heterogeneity in structure and extracellular matrix composition along the length of the tendon affects the biomechanical properties both locally and as a whole. In addition, activity levels can alter the viscoelastic properties of the tendon by increasing or decreasing tendon stiffness and ultimate stress/force (Maeda et al., 2011). In the mature tendon, chronic loading during physical training upregulates collagen turnover, perhaps leading to overall increased collagen synthesis, and improves the tendon’s ability to handle various stresses and loads over time (Kjaer, 2004). As described earlier, what constitutes repetitive overloading *in vivo* is not consistent as it is both temporally- and architecturally-dependent.

## 5 Model Systems

### 5.1 *Ex Vivo*

**FIGURE 5:**
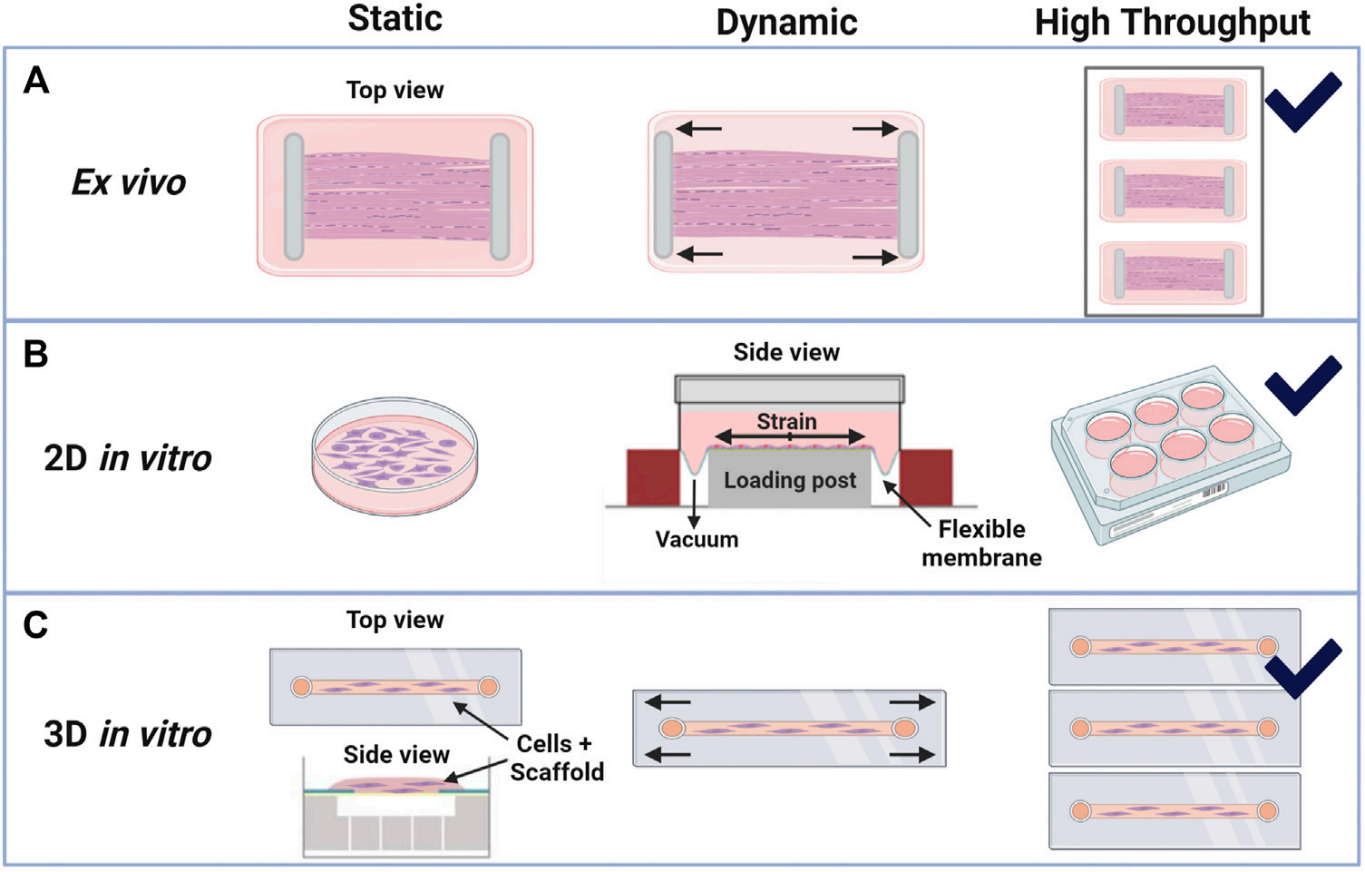
Bioreactor schematics for *in vitro* and *ex vivo* systems with static or dynamic loading and high-throughput options. Created with Biorender.com. **(A)** Example *ex vivo* systems are commonly developed in-house with whole tendon clamping systems that can be uniaxially stretched under set strains or loads (Wang et al., 2015; Tohidnezhad et al., 2020; Pedaprolu and Szczesny, 2021). **(B)** Tenocyte cell culture monolayers (2D *in vitro* systems) under static load, or no load, and dynamic loads have the greatest high-throughput capabilities. Dynamic loads on flexible membranes may be produced by mechanical actuators or with vacuum pressure, as illustrated and modified from Flexcell International’s Tension System, Burlington, NC (Arnoczky et al., 2002; Fleischhacker et al., 2020; Gaut et al., 2020; Kubo et al., 2020). **(C)** Cell-seeded 3D constructs can be gel-like, as shown in the side view (modified from Flexcell’s 3D Tissue Train System), or like whole-tissue if decellularized constructs are used. These 3D systems are generally bound by the dimensions of the material or well in which they are contained, yet there is flexibility in load types (tensile, shear, etc.) that can be analyzed (Zhang et al., 2015; Patel et al., 2017; Wang and Thien, 2018; Sawadkar et al., 2020; Pentzold and Wildemann, 2022).

Studies in *ex vivo* models have shown that cell apoptosis and upregulation of inflammatory pathways occurs at 9–20% strain loading values (Maeda et al., 2010; Thorpe et al., 2015; Wang et al., 2015). Tendon rupture also leads to massive tenocyte cell death through high expression of TGF-β (Maeda et al., 2011). Meanwhile, underloading-induced tendon degeneration is driven primarily by enzymatic digestion which affects the tendon mechanics (Maeda et al., 2011; Wang and Chen, 2018). For example, unloaded rabbit patellar tendons in the presence of collagenase have an 80% decrease in elongation to failure, max failure force, and linear stiffness as compared to tendons loaded at 4% static strain (Nabeshima et al., 1996). Only physiological dynamic loading, which is generally between 4–8% strain at 0.25–1 Hz in *ex vivo* models, leads to increased collagen production and regenerative properties (Legerlotz et al., 2013; Wang et al., 2013, 2015). Highlighted tendon mechanobiology *ex vivo* work is summarized in Table 1 for recent findings from years 2015–2021. In addition, a general schematic of the combined culture and loading systems (bioreactors) used for *ex vivo* studies included in this review are illustrated in Figure 5A.

**TABLE 1:** Highlighted summaries of recent *ex vivo* studies with associated loading schemes. Images created with Biorender.com.

Author/year	Type	Model	Results
Connizizzo and Grodzinsky. (2018)	*Ex vivo*	Mouse bone-tendon-muscle (supraspinatus)	Unloaded bone-tendon-muscle has greater pro-inflammatory cytokine expression and tenocyte apoptosis compared to tendon only, suggesting injured bone/muscle may exacerbate tendinopathic features.
Pedaprolu and Szczesny. (2021)	*Ex vivo*	Mouse Achilles tendon	Design and validation of an open-source bioreactor for tendon *ex vivo* studies that produce physiologically-relevant stress-based loading instead of strain-based loading protocols.
Wang et al. (2015)	*Ex vivo*	Rabbit Achilles tendon	Reversal of unloading-induced degenerative markers such as increased Col3 and apoptosis in tendon culture (day 6) through dynamic, physiological loading protocols (day 12).
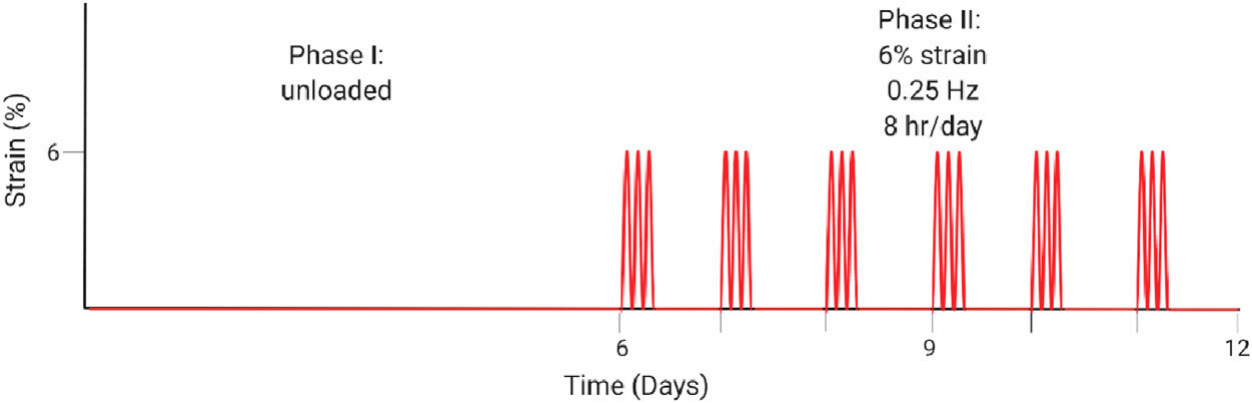			
Thorpe et al. (2015)	*Ex vivo*	Equine superdigital flexor tendon fascicles	Overloading at 12% cyclic strain for 30 min at 1 Hz vs. 2% static strain initiates tenocyte-driven inflammatory marker upregulation and matrix degradation, highlighting that inflammatory pathways also play a role in matrix degeneration.
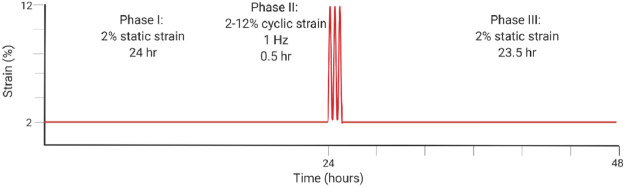			
Tohidnezhad et al. (2020)	*Ex vivo*	Rat flexor digitorum longus tendon	Greater expression of MMPs and proteoglycans in tendon gliding versus traction areas in response to dynamic loading. Indicates that various anatomies differentially respond to loading and likely differentially contribute to tendinopathies.
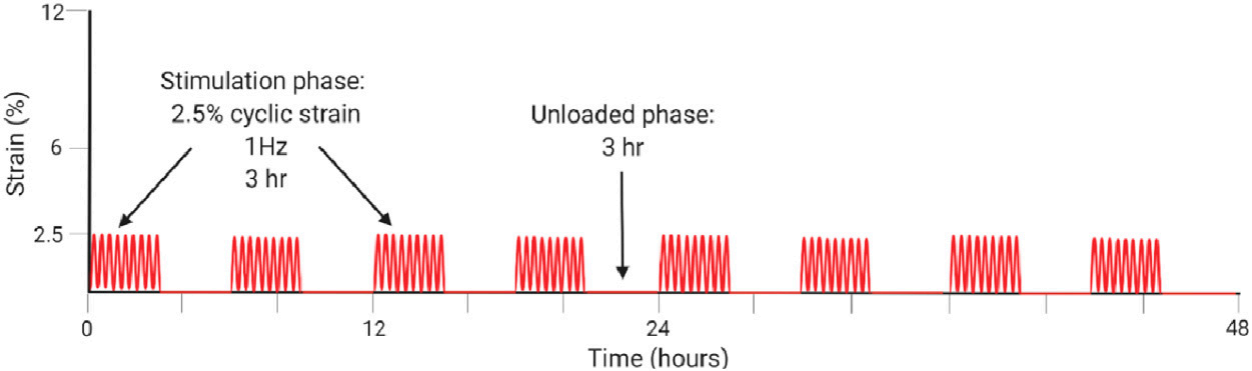			

Many of the previously described *ex vivo* studies were limited in analyzing longer-term inflammatory effects, especially in overloading of explants. Only Maeda et al., 2011, measured cytokine factors after 7 days of culture with no difference found between unloaded and physiologically loaded groups. Despite this finding, measuring pro-inflammatory factors in hyper-physiologically loaded tissues over at least a 7-day period may help elucidate pathomechanisms associated with tendon matrix modulation by removing other tissue effects. Downstream of TGF-β signaling, hyper-physiological loading is expected to increase IL-1β expression (Figure 2B). It would be helpful to characterize temporal IL-1β expression in hyper-physiological loading scenarios to identify potential feedback loops within the tissue.

Inflammatory cascades within tendon can originate from other sources (Arvind and Huang, 2021). In a rotator cuff muscle, bone, and tendon explant culture model, 7 days in culture results in upregulation of TNF-α and IL-6, atrophy, and tenocyte cell death as compared to the tendon only explant culture (Connizzo and Grodzinsky, 2018). These findings correlate with upregulation of TNF-α, interleukin-6 (IL-6), and IL-1 in bone injuries (Connizzo and Grodzinsky, 2018) and reinforce that unloaded tendon explants alone may produce limited quantities of pro-inflammatory factors (Maeda et al., 2011). This indicates that the muscle and bone likely play a role in the inflammatory signaling and ultimate degeneration of tendon after injury. Culture conditions, specifically serum-supplemented media, likely play a role in directing tenocytes to a pro-inflammatory phenotype as well in both whole tendon explants and 2D culture (van Vijven et al., 2021). Underlying presence of pro-inflammatory factors is generally unknown or varies in serum batches, which may contribute to the pro-inflammatory phenotypes. A primary limitation with these studies is that the tissues were unloaded once explanted; however, these results highlight the need to understand the role of other tissues and culture conditions in inflammatory signaling.

Mechanical loading to a specific strain value is less physiologically relevant as compared to stress-specific loading. This is due to the viscoelastic nature of the tendon, where repeated loading to a certain strain level on the tendon leads to decreasing absolute stress over time. Variations in cross-sectional area lead to differential load distribution across samples when stretched to the same strain as well. An *ex vivo* bioreactor was designed for this issue specifically, where researchers could characterize the link between tendon explant fatigue loading with tendon degeneration using cyclic loads and stresses instead of strains (Pedaprolu and Szczesny, 2021).

Physiological mechanical loading is necessary for tissue homeostasis though may also have therapeutic effects following injury. In a study by Wang et al., researchers induced early-stage tendinopathy of the rabbit Achilles tendon by unloading the tendon for 6 or 12 days, and then they reversed the effect in both treatment groups *via* external mechanical stimulation in a previously developed bioreactor (Wang et al., 2015). They stimulated the tendon under 6% strain, 0.25 Hz, and 8 h per day for 6 days based on their previous work estimating loads of 5–6% strain during hopping (schematic illustrated in Table 1). After the 6 days, results indicated reversal of the biochemical, structural, and mechanical changes that were induced by unloading. Significantly, they measured increased Col1 production and decreased cell apoptosis and ECM degradation (Wang et al., 2015). Their work highlights the need for further research on the regenerative capacity of tendon.

Overall, few studies have focused on gliding tendons and the role that compressive and shear forces play in tendon pathologies such as carpal tunnel syndrome (Tse and Keir, 2020). The pathomechanism is widely unknown for such cases. A pilot study by Tohidnezhad et al. measured tenocyte-specific marker expression and ECM remodeling components (MMP-1 and 13 and proteoglycans) in both the traction and gliding, with chondrocyte-like tenocytes, areas of the tendon after 48 hours of uniaxial cyclical loading (refer to Table 1 for the loading schematic). They found that the gliding area of the tendon that is exposed to greater shear and compressive forces had greater upregulation of matrix degradation markers compared to the traction area (Tohidnezhad et al., 2020). This may correlate to clinical observations that tendon ruptures frequently occur in the gliding area of tendons. However, limitations of this study include the short stimulation period (48 h) and limited analysis of the many factors involved in matrix remodeling.

### 5.2 *In Vitro*

Previous work indicates that loading type (biaxial versus uniaxial) and structural environment (2D versus 3D) affect cellular responses *in vitro*. Loading strains of greater than 10–12% induce expected pathways, such as MMP upregulation, inflammatory factors, cell apoptosis, and angiogenic factors in 2D (Arnoczky et al., 2002) and upregulation of inflammatory factors and MMP with decreased tenogenic factors in 3D cultures (Pentzold and Wildemann, 2022). Similarly, underloading led to activation of cellular inflammation and matrix degradation pathways (Arnoczky et al., 2002;; Tsuzaki et al., 2003a; Chen et al., 2009). Physiological loading ranges vary depending if the platform is 2D or 3D as well as 3D construct material (Patel et al., 2017). In general, local strains to the cells in 3D culture are dependent on the scaffold material and crosslinking density of hydrogels (Bryant et al., 2004). Fibrous scaffolds also may lead to higher variability than bulk 3D hydrogels that can more uniformly transfer loads to all cells within the construct (Kloxin et al., 2010). Highlighted tendon mechanobiology *in vitro* work is summarized in Table 2 for recent findings from years 2015–2020. Bioreactor setups used for *in vitro* work are outlined in Figures 5B,C.

**TABLE 2:** Highlighted summaries of recent *in vitro* studies with associated loading schemes. Images created with Biorender.com.

Author/year	Type	Model	Results
Kubo et al. (2020)	*In vitro*	Mouse Achilles tendon tenocytes in 2D culture	Tenocytes are sensitive and responsive to loading frequency in 2D culture, where 1 Hz frequency induces greater anabolic metabolism of ECM components and greater overall proliferation as compared to loading at 2 Hz.
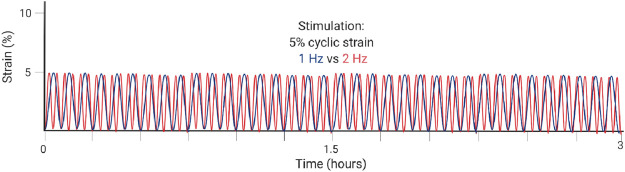			
Patel et al. (2017)	*In vitro*	Peptide polyethylene glycol hydrogel of rat tail tendon, and bovine and equine flexor and extensor tenocytes	Compared fiber composite techniques for 3D biomimetic hydrogels that induce tenocyte expression of inhibitors of matrix degrading enzymes and that optimize the local force values, including shear force, that match local micromechanics of various whole tendon explant sources.
Sawadkar et al. (2020)	*In vitro*	Collagen 3D hydrogels	Cyclical uniaxial loading induced expected matrix modulation markers in tenocytes suspended in 3D hydrogels compared to static or unloaded controls.
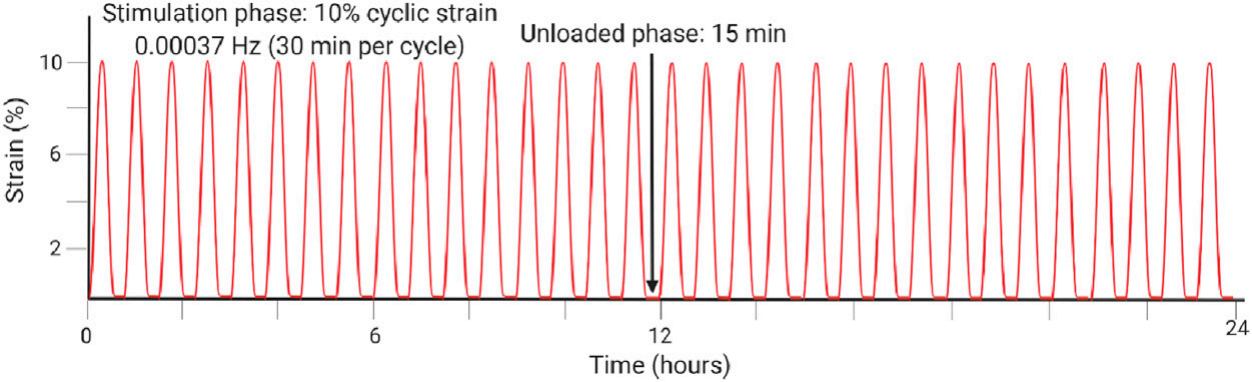			
Stolk et al. (2017)	*In vitro*	Human supraspinatus tenocyte and activated macrophage co-culture	Tenocytes pre-stimulated with mixed pro-inflammatory cytokines for 3 days, then co-cultured with activated macrophages for 3 days alter their surface markers and lead to greater pro-inflammatory macrophage polarization compared to unstimulated tenocytes. Suggests potential role of tenocyte and macrophage crosstalk in the perpetuation of altered healing responses that is characteristic of tendinopathy.
Zhang et al. (2015)	*In vitro*	Injured mouse Achilles tendon TPCs	Injured tendon progenitor cell (TPC) is shifted to a pro-inflammatory metabolic state and its tenogenic differentiation potential is permanently inhibited by the presence of IL-1β *in vitro*, indicating IL-1β′s potential role in tendinopathic features.

For *in vitro* studies, loading type and frequency affect catabolic and anabolic pathways. In 2D, a frequency of 1 Hz (similar to walking) compared to 2 Hz (similar to sprinting) lead to greater cell proliferation of 2D cultures, greater cell viability, greater Col 1 expression, reduced expression of MMP-1 and MMP-13, and decreased expression of VEGF (Kubo et al., 2020). The loading schematic is illustrated in Table 2 for Kubo et al., 2020. In 3D cell constructs, dynamic loading type affects TPC differentiation and tendon formation *via* altering pathway activation. With biaxial loading, tenocytes experience both longitudinal and radial or lateral loading. Uniaxial loading provides a more physiologically relevant stimulation of the 3D cell culture niche that is necessary for tenogenic differentiation (Wang and Thien, 2018). Uniaxial loading in 3D cell culture led to the discovery that the transmembrane integrin protein is activated *via* the PI3K/AKT pathway (Manning and Cantley, 2007; Paterno et al., 2011). Meanwhile, tissue constructs aim to mimic the collagen fibers loading scheme with the correct ratio of higher magnitude tensile to lower magnitude shear forces (Patel et al., 2017; Sawadkar et al., 2020). In native tendon, whole tissue strain varies from the local fiber strains, with fiber extension reaching only about 40% of the whole tissue (Thorpe et al., 2013; Shepherd et al., 2014). One particular 3D hydrogel was developed to mimic the dynamics of collagen fibers in the native tendon and therefore forces, such that the sliding of fibers produced shear forces and fiber extension as tension (Patel et al., 2017).

As with *ex vivo* models, there is a need to characterize inflammatory effects in these models of tendon degeneration. Studies found that IL-1β expression is upregulated in tendon cells by exogenous IL-1β (M Tsuzaki et al., 2003b), which triggers the cytokine-induced MMP matrix degradation pathways due to fibroblast activation. In addition, IL-1β alters metabolic pathways and inhibits TPC differentiation potential irreversibly through downregulation of Col1, Col3, *Scx*, *Tnmd*, and biglycan (Zhang et al., 2015). Another pilot study by Stolk et al. highlighted that pro-inflammatory pre-stimulated human tenocytes co-cultured with macrophages lead to altered surface markers and increased expression of inflammatory cytokines, such as IL-6 and IL-8, as well as influenced macrophage polarization to a pro-inflammatory state after 3 days (Stolk et al., 2017). Results from this work corroborates cytokine signals originating from other tissues might play a role in tendon degenerative pathways as well as contribute to a positive-feedback loop in tendon cells.

Although 3D cultures facilitate manipulation of the loading protocols, the current limitation of this work is that limited inflammatory analysis is performed on tissue constructs. More specifically, combinatory effects of loading and culture conditions on inflammatory responses needs to be explored. Determining inflammatory effects *in vitro* is also further complicated by tenocyte response variations associated with the model species used (Oreff et al., 2021), which indicates the importance of selecting a model that most appropriately matches the research question.

### 5.3 Other Model Systems

Live animal models are commonly used in the study of tendinopathy as they are the most physiologically relevant. Overloading of the Achilles tendons is often performed in rodents as they have homologous anatomy and physiology to humans. They have shown that intensive treadmill running induces degenerative tendon pathologies, including TPC differentiation into chondrocyte-like cells that lead to non-tendinous tissue signaling (Zhang et al., 2020). Meanwhile, more moderate treadmill running promotes TPC differentiation into tenocytes that achieve homeostasis (Zhang et al., 2020). In a recent equine model, researchers found that biomechanical and compositional adaptation that occur from mechanical loading are localized to the non-collagenous extracellular matrix (Zamboulis et al., 2020). However, discrepancies may exist between *in vivo* and *in vitro* models. A pilot study illustrated differences between expressions of ECM components for *in vitro* mouse Achilles tenocytes versus *in vivo* mouse Achilles tendons. Specifically, *in vitro* loading led to a higher expression of collagen type III versus *in vivo* (Fleischhacker et al., 2020). Though successful at modeling tendinopathy, *in vivo* models are plagued by confounding variables compounded by the difficulty in identifying the underlying mechanisms.

Nevertheless, *in vivo* models highlight important findings that could shift focus for future investigations, as presented by Zamboulis et al., 2020 with the finding of non-collagenous matrix adaptations. A piezo-bioelectric device used with *in vitro* and *in vivo* systems of tendon regeneration suggest the need to further investigate the role of mechanical stimulation in modulating tenogenic-specific phenotypes (Fernandez-Yague et al., 2021). Fernandez-Yague *et al.* suggests that mechanical stimulation may induce pro-regenerative pathways *via* modulation of ion channel sensitivity. Furthermore, findings from Passini *et al.* suggest that shear stress is sensed through the specific PIEZO1 calcium ion channel in rodents and leads to upregulation of collagen cross-linking factors related to tendon stiffness (Passini et al., 2021). The direct effect of various mechanical stimuli in modulating these mechanosensing pathways is important to uncover in future work.

Overall, live models of tendon degeneration and regeneration show age-dependent mechanistic effects (Freedman et al., 2022; Kinitz et al., 2022). Specifically, mouse Achilles tendon following transection recruits *Scx-lineage* tenocytes to the repair site in neonatal development, but not in adult mice (Howell et al., 2017). The neonatal healing strategy ultimately results in functional restoration, whereas the adult suffers from dysfunction due to fibrotic scarring. Despite this new finding, the underlying mechanism of *Scx-lineage* tenocyte recruitment is unclear.

Recent advancements in multiscale models have enabled researchers to estimate *in vivo* stress and strain values (Pizzolato et al., 2020; Devaprakash et al., 2022) as well as investigate the role of mechanical loading (both quasi-static and dynamic) and tendon healing on multiscale mechanical, structural, and compositional properties (Freedman et al., 2018). Patient-specific finite element (FE) models of the human Achilles tendon and an OpenSim neuromusckuloskeletal model may be combined for measuring real-time stress and strain values during various activities (Pizzolato et al., 2020). In another FE analysis, macroscale tendon strain stiffening is reduced with higher magnitude and longer duration loading as well as increased laxity and delayed fiber re-alignment with applied strain (Freedman et al., 2018). Microscale properties during early healing were found to differ greatly from uninjured tendon. Predicted deficits in ECM stress transmission were found following fatigue loading and during healing in FE analysis (Freedman et al., 2018). In general, FE analysis allows for rapid testing that can facilitate experimental designs without the use of live animals, but it also may aid in patient-specific tendon rehabilitation protocols.

### 5.4 Evaluation of Model Systems

Various model systems are utilized to study tendon mechanobiology. As shown in Table 3, key features of each type of model system (*ex vivo*, 2D *in vitro*, 3D *in vitro*, and *in vivo*) are semi-quantitatively rated on their overall ability to replicate seven desired features when performing assessments of tendon mechanobiology. These key features include “biological relevance”, “biological controllability”, “biomechanical relevance”, “biomechanical controllability”, “accessibility”, “usability”, and “translatability”. Only mammalians are included in this assessment as they share similar immunological characteristics to humans. Key feature rating values were assigned to the model system types on a relative scale of one to four due to the four types of model systems that are being evaluated. For instance, a value of “one” for “biological relevance” for 2D *in vitro* systems was assigned as it least mimics the physiological environment as compared to 3D *in vitro* (second lowest = 2), *ex vivo* (3), and *in vivo* (highest = 4).

**TABLE 3:** Summary chart of features desired by various model types for replicating and characterizing tendon mechanoresponses (scale: 1 = lowest to 4 = highest).

Desired feature	*Ex vivo*	*In vitro* (2D)	*In vitro* (3D)	*In vivo*
Biological relevance	3	1	2	4
Biological controllability	2	4	3	1
Biomechanical relevance	3	1	2	4
Biomechanical controllability	2	4	3	1
Accessibility	1/2	4	3	1/2
Usability	1	4	3	2
Translatability	3	1	2	4
Total Score	13/14	19	18	17/18

Each key feature has particular aspects that were considered in the rating assessment of the model systems though some of their ratings are similar. For “biological relevance”, the presence of other physiological cues was considered, such as immune and cell signaling crosstalk (2D lowest, *in vivo* highest). Because cells in 2D culture are isolated from other tissues and extended cell culture may alter cell phenotype, 2D *in vitro* systems rank the lowest for “biological relevance”. For “biological controllability”, the ability to control crosstalk factors was considered and the opposite rating trend was assigned (2D highest, *in vivo* lowest). Cultures in 2D have the greatest controllability as crosstalk can be isolated to specific cell types or factors using co-culturing techniques. Then cell-matrix interactions can be captured and manipulated with 3D systems, which is why 3D systems rank the second highest for “biological controllability”, followed by *ex vivo* (2) and *in vivo* (1) systems. Similarly, “biomechanical relevance” is related to the ability of the investigator to recapitulate the *in vivo* biomechanical environment (2D lowest, *in vivo* highest), while “biomechanical controllability” relates to the ability to control the mechanical loading environment (2D highest, *in vivo* lowest). Explanted tissues, or *ex vivo* models, maintain the native extracellular matrix, which affects mechanosensing pathways, and this contributes to its higher rating than both 2D and 3D *in vitro* systems for both biological and biomechanical relevance. *In vivo* models rank highest in biomechanical relevance because natural variation in loading parameters, gait, and load sharing with other tissues are not recapitulated in *ex vivo* systems.

Aspects for the other three key features are straightforward. The “accessibility” of a model system relates to the ability to obtain the desired tissue or animal sources for that system. Because primary cell lines are often the most accessible, 2D *in vitro* systems scored highest (4), followed by 3D *in vitro* (3), and then *ex vivo* and *in vivo* systems share a rating of 1 or 2 as access to whole tissue for *ex vivo* work or live animals for *in vivo* work may depend on the institution and regulations. Usability relates to the technical feasibility of developing the model system for dynamic loading experiments (2D highest, followed by 3D, *in vivo*, and then *ex vivo* systems). Development of the model system includes aspects such as accruing a bioreactor, troubleshooting, and experimental timelines. Again, these rankings might be institution- or resource-dependent. Last, translatability relates to how well these model systems translate to clinical work, where 2D *in vitro* has the lowest level of translation while *in vivo* systems are the most translatable.

In this assessment, each key feature was equally weighted to minimize subjective evaluation; however, this weighting could change based on the research question. For example, “biomechanical controllability” would likely be weighted more heavily than “biomechanical relevance” if an investigator was aiming to determine underlying cell signaling factors for varied loading conditions.

## 6 Discussion

Mechanical loading of tendon tissue is essential for tendon maturation during development, tendon homeostasis, and degeneration. Many reviews focus in-depth on the history of understanding matrix turnover, tendon biomechanics, and the methods/models used to understand tendon as well as ligament mechanobiology (Lavagnino et al., 2015; Thomopoulos et al., 2015; Wang and Chen, 2018; Dyment et al., 2020; Friese et al., 2020; Gracey et al., 2020; Wang et al., 2020; Bramson et al., 2021). In addition, *in vivo* models of tendon degeneration are the focus of another review (Theodossiou and Schiele, 2019). This review summarizes the more recent findings relating to tendon mechanobiology using various model systems. In addition, it provides an overall assessment or rating of model systems in their ability to replicate various key features necessary to evaluate tendon mechanobiology. This method of assessing the propriety of a system may facilitate experimental design as it can be tailored for the targeted research question. Based on this assessment, the sum total for 2D *in vitro* systems was greatest overall (19 out of a max of 28), followed by 3D *in vitro* (18), *in vivo* (17/18), and then *ex vivo* systems (13/14).

Though significant improvements in understanding inflammatory mediators in normal tendon healing have been achieved (Chisari et al., 2019; Arvind and Huang, 2021), inflammation is still not well understood as an effector in tendon pathology, especially its temporal role. For instance, co-culture studies show that tenocyte and macrophage crosstalk likely facilitates macrophage polarization to a pro-inflammatory state after 3 days (Stolk et al., 2017). Yet it is unclear when macrophages polarize to the inflammation-resolving state during healing, if inflammation is fully resolved in pathology, and if tenocytes play a role in this polarization at all. As such, it is necessary to tease apart the inflammatory crosstalk occurring between tissues and the immune system.

Future studies focused on longer-term experiments (Maeda et al., 2011) and decoupling mechanical loading and inflammation crosstalk is recommended to fully characterize model systems of tendon pathology. As it is common for many engineered 3D constructs under dynamic loads to physically weaken after only a few days, the use of explanted tissue or decellularized constructs may help mitigate the limitations associated with experiment length. In addition, decoupling mechanical effects alongside mechanosensing pathways may be accomplished with bioreactor designs that include various mechanical stimulation options, or perhaps through the use of piezo-electric devices in cultivation studies (Fernandez-Yague et al., 2021; Passini et al., 2021). Last, *in vivo* stress, strain, and frequency values should inform loading paradigms for *in vitro* and *ex vivo* bioreactor studies.

The ultimate goal of this work is to improve regenerative therapies and clinical outcomes, or prevent tendon pathologies entirely. Many studies have limited translation due to heterogeneous experimental designs, bioreactor systems, and loading schemes. Addressing these limitations is essential to progressing the field of tendon mechanobiology, though teasing apart the effects of inflammation from the associated mechanical stimulatory effects is not a simple task. Taken together, improvements of *in vitro*, *ex vivo*, and *in vivo* model systems could improve overall translation. Conclusively, considerable attention on performing dynamic loading studies of at least 7 days with inflammatory analysis is paramount due to the scarcity of such explorations currently in the literature.

## Abbreviations

ECM, extracellular matrix; TPC, tendon progenitor cell; Col 1, collagen type I; Col 3, collagen type III; MMP, matrix metalloproteinases; ADAMT, disintegrin and metalloproteinase with thrombospondin motifs; TGF-β, transforming growth factor β; *Mhk*, Mohawk; *Scx*, Scleraxis; *Tnmd*, Tenomodulin; IL-1β, interleukin-1β; TNF-α, tumor necrosis factor α; VEGF, vascular endothelial growth factor; IL-6, interleukin-6; FEA, finite elemental analysis.

## References

[B1] Abate M. Gravare-Silbernagel K. Siljeholm C. Di Iorio A. De Amicis D. Salini V. (2009). Pathogenesis of Tendinopathies: Inflammation or Degeneration? Arthritis Res. Ther. 11 (235), 235–315. 10.1186/AR2723 19591655 PMC2714139

[B2] Agarwal S. Loder S. J. Cholok D. Peterson J. Li J. Breuler C. (2017). Scleraxis-Lineage Cells Contribute to Ectopic Bone Formation in Muscle and Tendon. NIH Public Access 35 (3), 705–710. 10.1002/STEM.2515 PMC5529170 27862618

[B3] Alim M. A. Peterson M. Pejler G. (2020). Do mast Cells Have a Role in Tendon Healing and Inflammation? Cells 9 (5), 1134–1215. 10.3390/cells9051134 PMC7290807 32375419

[B4] Ansorge H. L. Adams S. Birk D. E. Soslowsky L. J. (2011). Mechanical, Compositional, and Structural Properties of the Post-natal Mouse Achilles Tendon. Ann. Biomed. Eng. 39 (7), 1904–1913. 10.1007/S10439-011-0299-0 21431455 PMC3341172

[B5] Ansorge H. L. Hsu J. E. Edelstein L. Adams S. Birk D. E. Soslowsky L. J. (2012). Recapitulation of the Achilles Tendon Mechanical Properties during Neonatal Development: a Study of Differential Healing during Two Stages of Development in a Mouse Model. J. Orthop. Res. 30 (3), 448–456. 10.1002/JOR.21542 22267191 PMC3265027

[B6] Arnoczky S. P. Tian T. Lavagnino M. Gardner K. Schuler P. Morse P. (2002). Activation of Stress-Activated Protein Kinases (SAPK) in Tendon Cells Following Cyclic Strain: the Effects of Strain Frequency, Strain Magnitude, and Cytosolic Calcium. J. Orthop. Res. 20 (5), 947–952. 10.1016/S0736-0266(02)00038-4 12382958

[B7] Arvind V. Huang A. H. (2021). Reparative and Maladaptive Inflammation in Tendon Healing. Front. Bioeng. Biotechnol. 9, 1–16. 10.3389/FBIOE.2021.719047/BIBTEX PMC8327090 34350166

[B8] Attia M. Scott A. Carpentier G. Lian Ø. Van Kuppevelt T. Gossard C. (2014). Greater Glycosaminoglycan Content in Human Patellar Tendon Biopsies Is Associated with More Pain and a Lower VISA Score. Br. J. Sports Med. 48 (6), 469–475. 10.1136/BJSPORTS-2013-092633 24100290

[B9] Berthet E. Chen C. Butcher K. Schneider R. A. Alliston T. Amirtharajah M. (2013). Smad3 Binds Scleraxis and Mohawk and Regulates Tendon Matrix Organization. J. Orthop. Res. 31 (9), 1475–1483. 10.1002/JOR.22382 23653374 PMC3960924

[B10] Birk D. E. Mayne R. (1997). Localization of Collagen Types I, III and V during Tendon Development. Changes in Collagen Types I and III Are Correlated with Changes in Fibril Diameter. Eur. J. Cell. Biol. 72, 352–361. 9127735

[B11] Bramson M. T. K. Van Houten S. K. Corr D. T. (2021). Mechanobiology in Tendon, Ligament, and Skeletal Muscle Tissue Engineering. J. Biomechanical Eng. 143 (7), 1–15. 10.1115/1.4050035/1097189 33537704

[B12] Bryant S. J. Chowdhury T. T. Lee D. A. Bader D. L. Anseth K. S. (2004)., 32. CA, 407–417. 10.1023/B:ABME.0000017535.0060210.1023/b:abme.0000017535.00602.ca Crosslinking Density Influences Chondrocyte Metabolism in Dynamically Loaded Photocrosslinked Poly(ethylene Glycol) Hydrogels Ann. Biomed. Eng. 15095815

[B13] Buono A. D. Oliva F. Osti L. Maffulli N. (2013). Metalloproteases and Tendinopathy. Mltj 3 (1), 51–57. 10.11138/MLTJ/2013.3.1.051 23885345 PMC3676164

[B14] Burgio V. Civera M. Rodriguez Reinoso M. Pizzolante E. Prezioso S. Bertuglia A. (2022). Mechanical Properties of Animal Tendons: A Review and Comparative Study for the Identification of the Most Suitable Human Tendon Surrogates. Processes 10 (485), 485–520. 10.3390/PR10030485

[B15] Burk J. (2019). “Mechanisms of Action of Multipotent Mesenchymal Stromal Cells in Tendon Disease,” in Tendons. Editor Sözen H. (London: IntechOpen), 1–31. 10.5772/INTECHOPEN.83745

[B16] Chen Y.-J. Huang C.-H. Lee I.-C. Lee Y.-T. Chen M.-H. Young T.-H. (2009). Effects of Cyclic Mechanical Stretching on the mRNA Expression of Tendon/ligament-Related and Osteoblast-specific Genes in Human Mesenchymal Stem Cells. Connect. Tissue Res. 49 (1), 7–14. 10.1080/03008200701818561 18293173

[B17] Chisari E. Rehak L. Khan W. S. Maffulli N. (2019). Tendon Healing in Presence of Chronic Low-Level Inflammation: a Systematic Review. Br. Med. Bull. 132 (1), 97–116. 10.1093/BMB/LDZ035 31838495

[B18] Connizzo B. K. Grodzinsky A. J. (2018). Release of Pro-inflammatory Cytokines from Muscle and Bone Causes Tenocyte Death in a Novel Rotator Cuff *In Vitro* Explant Culture Model. Connect. Tissue Res. 59 (5), 423–436. 10.1080/03008207.2018.1439486 29447021 PMC6240787

[B19] Cosgrove B. D. Mui K. L. Driscoll T. P. Caliari S. R. Mehta K. D. Assoian R. K. (2016). N-Cadherin Adhesive Interactions Modulate Matrix Mechanosensing and Fate Commitment of Mesenchymal Stem Cells. Nat. Mater 15 (12), 1297–1306. 10.1038/nmat4725 27525568 PMC5121068

[B20] Dahlgren L. A. Mohammed H. O. Nixon A. J. (2005). Temporal Expression of Growth Factors and Matrix Molecules in Healing Tendon Lesions. J. Orthop. Res. 23 (1), 84–92. 10.1016/j.orthres.2004.05.007 15607879

[B21] Dakin S. G. Buckley C. D. Al-Mossawi M. H. Hedley R. Martinez F. O. Wheway K. (2017). Persistent Stromal Fibroblast Activation Is Present in Chronic Tendinopathy. Arthritis Res. Ther. 19 (16), 1–11. 10.1186/S13075-016-1218-4 28122639 PMC5264298

[B22] Dakin S. G. Werling D. Hibbert A. Abayasekara D. R. E. Young N. J. Smith R. K. W. (2012). Macrophage Sub-populations and the Lipoxin A4 Receptor Implicate Active Inflammation during Equine Tendon Repair. PLoS ONE 7 (2), e32333–12. 10.1371/JOURNAL.PONE.0032333 22384219 PMC3284560

[B23] Devaprakash D. Graham D. F. Barrett R. S. Lloyd D. G. Obst S. J. Kennedy B. (2022). Free Achilles Tendon Strain during Selected Rehabilitation, Locomotor, Jumping, and Landing Tasks. J. Appl. Physiology 132 (4), 956–965. 10.1152/japplphysiol.00662.2021 35142563

[B24] Dyment N. A. Barrett J. G. Awad H. A. Bautista C. A. Banes A. J. Butler D. L. (2020). A Brief History of Tendon and Ligament Bioreactors: Impact and Future Prospects. J. Orthop. Res. 38 (11), 2318–2330. 10.1002/JOR.24784 32579266 PMC7722018

[B25] Eliasson P. Fahlgren A. Pasternak B. Aspenberg P. (2007). Unloaded Rat Achilles Tendons Continue to Grow, but Lose Viscoelasticity. J. Appl. Physiology 103 (2), 459–463. 10.1152/japplphysiol.01333.2006 17412787

[B26] Eyre D. R. Paz M. A. Gallop P. M. (1984). Cross-linking in Collagen and Elastin. Annu. Rev. Biochem. 53 (1), 717–748. 10.1146/annurev.bi.53.070184.003441 6148038

[B27] Fernandez‐Yague M. A. Trotier A. Demir S. Abbah S. A. Larrañaga A. Thirumaran A. (2021). A Self‐Powered Piezo‐Bioelectric Device Regulates Tendon Repair‐Associated Signaling Pathways through Modulation of Mechanosensitive Ion Channels. Adv. Mater. 33 (40), 2008788–2008818. 10.1002/ADMA.202008788 PMC11468587 34423493

[B28] Fessel G. Snedeker J. G. (2009). Evidence against Proteoglycan Mediated Collagen Fibril Load Transmission and Dynamic Viscoelasticity in Tendon. Matrix Biol. 28 (8), 503–510. 10.1016/j.matbio.2009.08.002 19698786

[B29] Fleischhacker V. Klatte-Schulz F. Minkwitz S. Schmock A. Rummler M. Seliger A. (2020). *In Vivo* and *In Vitro* Mechanical Loading of Mouse Achilles Tendons and Tenocytes-A Pilot Study. Ijms 21 (1313), 1313–1315. 10.3390/ijms21041313 PMC7072865 32075290

[B30] Fleming D. M. Cross K. W. Barley M. A. (2005). Recent Changes in the Prevalence of Diseases Presenting for Health Care. Br. J. Gen. Pract. 55 (517), 589–595. 16105366 PMC1463227

[B31] Freedman B. R. Knecht R. S. Tinguely Y. Eskibozkurt G. E. Wang C. S. Mooney D. J. (2022). Aging and Matrix Viscoelasticity Affect Multiscale Tendon Properties and Tendon Derived Cell Behavior. Acta Biomater. 143 (4), 63–71. 10.1016/J.ACTBIO.2022.03.006 35278685 PMC11069350

[B32] Freedman B. R. Rodriguez A. B. Leiphart R. J. Newton J. B. Ban E. Sarver J. J. (2018). Dynamic Loading and Tendon Healing Affect Multiscale Tendon Properties and ECM Stress Transmission. Sci. Rep. 8, 1–13. 10.1038/s41598-018-29060-y 30022076 PMC6052000

[B33] Friese N. Gierschner M. B. Schadzek P. Roger Y. Hoffmann A. (2020). Regeneration of Damaged Tendon-Bone Junctions (Entheses)-TAK1 as a Potential Node Factor. Ijms 21 (15), 5177–5221. 10.3390/ijms21155177 PMC7432881 32707785

[B34] Fu S. C. Rolf C. Cheuk Y. C. Lui P. P. Chan K. M. (2010). Deciphering the Pathogenesis of Tendinopathy: a Three-Stages Process. Sports Med. Arthrosc. Rehabil. Ther. Technol. 2 (30), 30–12. 10.1186/1758-2555-2-30/TABLES/1 21144004 PMC3006368

[B35] Fu S.-C. Chan K.-M. Rolf C. G. (2007). Increased Deposition of Sulfated Glycosaminoglycans in Human Patellar Tendinopathy. Clin. J. Sport Med. 17 (2), 129–134. 10.1097/JSM.0b013e318037998f 17414481

[B36] Gaut L. Bonnin M.-A. Blavet C. Cacciapuoti I. Orpel M. Mericskay M. (2020). Mechanical and Molecular Parameters that Influence the Tendon Differentiation Potential of C3H10T1/2 Cells in 2D- and 3D-Culture Systems. Biol. Open 9 (2), 1–13. 10.1242/bio.047928 PMC6994949 31941700

[B37] Goodship A. E. Birch H. L. Wilson A. M. (1994). The Pathobiology and Repair of Tendon and Ligament Injury. Veterinary Clin. N. Am. Equine Pract. 10 (2), 323–349. 10.1016/S0749-0739(17)30359-0 7987721

[B38] Gracey E. Burssens A. Cambré I. Schett G. Lories R. McInnes I. B. (2020). Tendon and Ligament Mechanical Loading in the Pathogenesis of Inflammatory Arthritis. Nat. Rev. Rheumatol. 16 (4), 193–207. 10.1038/s41584-019-0364-x 32080619 PMC7815340

[B39] Howell K. Chien C. Bell R. Laudier D. Tufa S. F. Keene D. R. (2017). Novel Model of Tendon Regeneration Reveals Distinct Cell Mechanisms Underlying Regenerative and Fibrotic Tendon Healing. Sci. Rep. 7 (45238), 1–14. 10.1038/SREP45238 28332620 PMC5362908

[B40] Itoi E. Berglund L. J. Grabowski J. J. Schultz F. M. Growney E. S. Morrey B. F. (1995). Tensile Properties of the Supraspinatus Tendon. J. Orthop. Res. 13 (4), 578–584. 10.1002/jor.1100130413 7674074

[B41] Järvinen T. A. H. Järvinen T. L. N. Kannus P. ozsa L. Järvinen M. (2004) ‘Collagen Fibres of the Spontaneously Ruptured Human Tendons Display Decreased Thickness and Crimp Angle’, J. Orthop. Res., 22(6), pp. 1303–1309. 10.1016/j.orthres.2004.04.003 15475213

[B42] John T. Lodka D. Kohl B. Ertel W. Jammrath J. Conrad C. (2010). Effect of Pro-inflammatory and Immunoregulatory Cytokines on Human Tenocytes. J. Orthop. Res. 28 (8), a–n. 10.1002/JOR.21079 20127972

[B43] Johnson G. A. Tramaglini D. M. Levine R. E. Ohno K. Choi N.-Y. L-Y. Woo S. (1994). Tensile and Viscoelastic Properties of Human Patellar Tendon. J. Orthop. Res. 12 (6), 796–803. 10.1002/jor.1100120607 7983555

[B44] Józsa L. Kannus P. (1997). Histopathological Findings in Spontaneous Tendon Ruptures. Scand. J. Med. Sci. Sports 7 (2), 113–118. 10.1111/j.1600-0838.1997.tb00127.x 9211612

[B45] Kannus P. Józsa L. (1991). Histopathological Changes Preceding Spontaneous Rupture of a Tendon. A Controlled Study of 891 Patients. J. Bone & Jt. Surg. 73 (10), 1507–1525. 10.2106/00004623-199173100-00009 1748700

[B46] Kannus P. (2000). Structure of the Tendon Connective Tissue. Blackwell Munksgaard 10 (6), 312–320. 10.1034/J.1600-0838.2000.010006312.X 11085557

[B47] Khan K. M. Scott A. (2009). Mechanotherapy: How Physical Therapists' Prescription of Exercise Promotes Tissue Repair. Br. J. Sports Med. Assoc. Sport Excercise Med. 43 (4), 247–252. 10.1136/BJSM.2008.054239 PMC2662433 19244270

[B48] Kinitz R. Heyne E. Koch L. G. Britton S. L. Thierbach M. Wildemann B. (2022). The Effect of Age and Intrinsic Aerobic Exercise Capacity on the Expression of Inflammation and Remodeling Markers in Rat Achilles Tendons. Ijms 23 (79), 79–20. 10.3390/IJMS23010079 PMC8744822 35008516

[B49] Kjær M. (2004). Role of Extracellular Matrix in Adaptation of Tendon and Skeletal Muscle to Mechanical Loading. Physiol. Rev. 84 (2), 649–698. 10.1152/PHYSREV.00031.2003 15044685

[B50] Kloxin A. M. Tibbitt M. W. Anseth K. S. (2010). Synthesis of Photodegradable Hydrogels as Dynamically Tunable Cell Culture Platforms. Nat. Protoc. 5 (12), 1867–1887. 10.1038/nprot.2010.139.Synthesis 21127482 PMC3897936

[B51] Kubo Y. Hoffmann B. Goltz K. Schnakenberg U. Jahr H. Merkel R. (2020). Different Frequency of Cyclic Tensile Strain Relates to Anabolic/catabolic Conditions Consistent with Immunohistochemical Staining Intensity in Tenocytes. Ijms 21 (3), 1082–1114. 10.3390/IJMS21031082 PMC7037470 32041254

[B52] Kubow K. E. Vukmirovic R. Zhe L. Klotzsch E. Smith M. L. Gourdon D. (2015). Mechanical Forces Regulate the Interactions of Fibronectin and Collagen I in Extracellular Matrix. Nat. Commun. 6 (1), 1–11. 10.1038/ncomms9026 PMC4539566 26272817

[B53] Legerlotz K. Schjerling P. Langberg H. Brüggemann G.-P. Niehoff A. (2007). The Effect of Running, Strength, and Vibration Strength Training on the Mechanical, Morphological, and Biochemical Properties of the Achilles Tendon in Rats. J. Appl. Physiology 102 (2), 564–572. 10.1152/japplphysiol.00767.2006 17038489

[B54] Lavagnino M. Arnoczky S. P. Tian T. Vaupel Z. (2003). Effect of Amplitude and Frequency of Cyclic Tensile Strain on the Inhibition of MMP-1 mRNA Expression in Tendon Cells: an *In Vitro* Study. Connect. Tissue Res. 44 (3–4), 181–187. 10.1080/03008200390215881 14504039

[B55] Lavagnino M. Wall M. E. Little D. Banes A. J. Guilak F. Arnoczky S. P. (2015). Tendon mechanobiology:Current Knowledge and Future Research Opportunities. J. Orthop. Res. 33 (6), 813–822. 10.1002/JOR.22871 25763779 PMC4524513

[B56] Leéjard V. Brideau G. Blais F. Salingcarnboriboon R. Wagner G. Roehrl M. H. A. (2007). Scleraxis and NFATc Regulate the Expression of the Pro-α1(I) Collagen Gene in Tendon Fibroblasts. J. Biol. Chem. 282 (24), 17665–17675. 10.1074/JBC.M610113200 17430895

[B57] Legerlotz K. Jones G. C. Screen H. R. C. Riley G. P. (2013). Cyclic Loading of Tendon Fascicles Using a Novel Fatigue Loading System Increases Interleukin‐6 Expression by Tenocytes. Scand. J. Med. Sci. Sports 23 (1), 31–37. 10.1111/j.1600-0838.2011.01410.x 22092479 PMC3558793

[B58] Lipman K. Wang C. Ting K. Soo C. Zheng Z. (2018). Tendinopathy: Injury, Repair, and Current Exploration. Dddt Vol. 12, 591–603. 10.2147/DDDT.S154660 PMC5865563 29593382

[B59] Liu S. H. Yang R. S. al-Shaikh R. Lane J. M. (1995). Collagen in Tendon, Ligament, and Bone Healing. A Current Review. Clin. Orthop. Relat. Res. 1 (318), 265–278. 7671527

[B60] Lorimer A. V. Hume P. A. (2014). Achilles Tendon Injury Risk Factors Associated with Running. Sports Med. 44 (10), 1459–1472. 10.1007/s40279-014-0209-3 24898814

[B61] Lorimer A. V. Hume P. A. (2016). Stiffness as a Risk Factor for Achilles Tendon Injury in Running Athletes. Sports Med. 46 (12), 1921–1938. 10.1007/s40279-016-0526-9 27194434

[B62] Louis-Ugbo J. Leeson B. Hutton W. C. (2004). Tensile Properties of Fresh Human Calcaneal (Achilles) Tendons. Clin. Anat. 17 (1), 30–35. 10.1002/ca.10126 14695585

[B63] Maeda E. Fleischmann C. Mein C. A. Shelton J. C. Bader D. L. Lee D. A. (2010). Functional Analysis of Tenocytes Gene Expression in Tendon Fascicles Subjected to Cyclic Tensile Strain. Connect. Tissue Res. 51 (6), 434–444. 10.3109/03008201003597056 20497018

[B64] Maeda T. Sakabe T. Sunaga A. Sakai K. Rivera A. L. Keene D. R. (2011). Conversion of Mechanical Force into TGF-β-Mediated Biochemical Signals. Curr. Biol. 21 (11), 933–941. 10.1016/J.CUB.2011.04.007 21600772 PMC3118584

[B65] Maganaris C. N. Paul J. P. (1999). *In Vivo* human Tendon Mechanical Properties. J. Physiology 521 (1), 307–313. 10.1111/j.1469-7793.1999.00307.x PMC2269645 10562354

[B66] Manning B. D. Cantley L. C. (2007). AKT/PKB Signaling: Navigating Downstream. Cell. 129 (7), 1261–1274. 10.1016/J.CELL.2007.06.009 17604717 PMC2756685

[B67] Matson A. Konow N. Miller S. Konow P. P. Roberts T. J. (2012). Tendon Material Properties Vary and Are Interdependent Among turkey Hindlimb Muscles. J. Exp. Biol. 215 (20), 3552–3558. 10.1242/jeb.072728 22771746 PMC3459684

[B68] Mendias C. L. Gumucio J. P. Lynch E. B. (2012). Mechanical Loading and TGF-β Change the Expression of Multiple miRNAs in Tendon Fibroblasts. J. Appl. Physiology 113 (1), 56–62. 10.1152/JAPPLPHYSIOL.00301.2012 PMC3404830 22539168

[B69] Midwood K. S. Chiquet M. Tucker R. P. Orend G. (2016). Tenascin-C at a Glance. J. Cell. Sci. 129 (23), 4321–4327. 10.1242/JCS.190546 27875272

[B70] Munger J. S. Sheppard D. (2011). Cross Talk Among TGF- Signaling Pathways, Integrins, and the Extracellular Matrix. Cold Spring Harb. Perspect. Biol. 3 (11), a005017. 10.1101/CSHPERSPECT.A005017 21900405 PMC3220354

[B71] Nabeshima Y. Grood E. S. Sakurai A. Herman J. H. (1996). Uniaxial Tension Inhibits Tendon Collagen Degradation by Collagenasein Vitro. J. Orthop. Res. 14 (1), 123–130. 10.1002/JOR.1100140120 8618154

[B72] Oreff G. L. Fenu M. Vogl C. Ribitsch I. Jenner F. (2021). Species Variations in Tenocytes' Response to Inflammation Require Careful Selection of Animal Models for Tendon Research. Sci. Rep. 11 (1), 1–14. 10.1038/S41598-021-91914-9 34127759 PMC8203623

[B73] Passini F. S. Jaeger P. K. Saab A. S. Hanlon S. Chittim N. A. Arlt M. J. (2021). Shear-stress Sensing by PIEZO1 Regulates Tendon Stiffness in Rodents and Influences Jumping Performance in Humans. Nat. Biomed. Eng. 5 (12), 1457–1471. 10.1038/s41551-021-00716-x 34031557 PMC7612848

[B74] Patel D. Sharma S. Bryant S. J. Screen H. R. C. (2017). Recapitulating the Micromechanical Behavior of Tension and Shear in a Biomimetic Hydrogel for Controlling Tenocyte Response. Adv. Healthc. Mat. 6 (4), 1601095–1601097. 10.1002/ADHM.201601095 PMC5469035 28026126

[B75] Paterno J. Vial I. N. Wong V. W. Rustad K. C. Sorkin M. Shi Y. (2011). Akt-mediated Mechanotransduction in Murine Fibroblasts during Hypertrophic Scar Formation. Wound Repair Regen. 19 (1), 49–58. 10.1111/J.1524-475X.2010.00643.X 21134033

[B76] Pedaprolu K. Szczesny S. E. (2021). A Novel, Open Source, Low-Cost Bioreactor for Load-Controlled Cyclic Loading of Tendon Explants. bioRxiv. 10.1101/2021.03.16.435688 35147179

[B77] Pentzold S. Wildemann B. (2022). Mechanical Overload Decreases Tenogenic Differentiation Compared to Physiological Load in Bioartificial Tendons. J. Biol. Eng. 16 (5), 1–10. 10.1186/S13036-022-00283-Y 35241113 PMC8896085

[B78] Pizzolato C. Shim V. B. Lloyd D. G. Devaprakash D. Obst S. J. Newsham-West R. (2020). Targeted Achilles Tendon Training and Rehabilitation Using Personalized and Real-Time Multiscale Models of the Neuromusculoskeletal System. Front. Bioeng. Biotechnol. 8, 1–15. 10.3389/FBIOE.2020.00878 32903393 PMC7434842

[B79] Riley G. P. (2005). Gene Expression and Matrix Turnover in Overused and Damaged Tendons. Scand. J. Med. Sci. Sports 15 (4), 241–251. 10.1111/j.1600-0838.2005.00456.x 15998341

[B80] Robinson W. H. Lepus C. M. Wang Q. Raghu H. Mao R. Lindstrom T. M. (2016). Low-grade Inflammation as a Key Mediator of the Pathogenesis of Osteoarthritis. Nat. Rev. Rheumatol. 12 (10), 580–592. 10.1038/NRRHEUM.2016.136 27539668 PMC5500215

[B81] Sawadkar P. Player D. Bozec L. Mudera V. (2020). The Mechanobiology of Tendon Fibroblasts under Static and Uniaxial Cyclic Load in a 3D Tissue Engineered Model Mimicking Native Extracellular Matrix. J. Tissue Eng. Regen. Med. 14 (1), 135–146. 10.1002/term.2975 31622052

[B82] Schulze-Tanzil G. Al-Sadi O. Wiegand E. Ertel W. Busch C. Kohl B. (2011). The Role of Pro-inflammatory and Immunoregulatory Cytokines in Tendon Healing and Rupture: New Insights. Scand. J. Med. Sci. Sports 21 (3), 337–351. 10.1111/J.1600-0838.2010.01265.X 21210861

[B83] Screen H. R. C. Berk D. E. Kadler K. E. Ramirez F. Young M. F. (2015). Tendon Functional Extracellular Matrix. J. Orthop. Res. 33 (6), 793–799. 10.1002/jor.22818 25640030 PMC4507431

[B84] Screen H. R. C. Shelton J. C. Bader D. L. Lee D. A. (2005). Cyclic Tensile Strain Upregulates Collagen Synthesis in Isolated Tendon Fascicles. Biochem. Biophysical Res. Commun. 336 (2), 424–429. 10.1016/J.BBRC.2005.08.102 16137647

[B85] Shepherd J. H. Riley G. P. Screen H. R. C. (2014). Early Stage Fatigue Damage Occurs in Bovine Tendon Fascicles in the Absence of Changes in Mechanics at Either the Gross or Micro-structural Level. J. Mech. Behav. Biomed. Mater. 38 (100), 163–172. 10.1016/J.JMBBM.2014.06.005 25001495 PMC4148183

[B86] Stauber T. Blache U. Snedeker J. G. (2020). Tendon Tissue Microdamage and the Limits of Intrinsic Repair. Matrix Biol. 85-86, 68–79. 10.1016/j.matbio.2019.07.008 31325483

[B87] Stolk M. Klatte-Schulz F. Schmock A. Minkwitz S. Wildemann B. Seifert M. (2017). New Insights into Tenocyte-Immune Cell Interplay in an *In Vitro* Model of Inflammation. Sci. Rep. 7 (1), 1–14. 10.1038/s41598-017-09875-x 28851983 PMC5575127

[B88] Subramanian G. Stasuk A. Elsaadany M. Yildirim-Ayan E. (2017). Effect of Uniaxial Tensile Cyclic Loading Regimes on Matrix Organization and Tenogenic Differentiation of Adipose-Derived Stem Cells Encapsulated within 3D Collagen Scaffolds. Stem Cells Int. 2017, 1–16. 10.1155/2017/6072406 PMC5742457 29375625

[B89] Thampatty B. P. Wang J. H.-C. (2018). Mechanobiology of Young and Aging Tendons: *In Vivo* Studies with Treadmill Running. J. Orthop. Res. 36 (2), 557–565. 10.1002/jor.23761 28976604 PMC5839954

[B90] Theodossiou S. K. Pancheri N. M. Martes A. C. Bozeman A. L. Brumley M. R. Raveling A. R. (2021). Neonatal Spinal Cord Transection Decreases Hindlimb Weight-Bearing and Affects Formation of Achilles and Tail Tendons. J. Biomech. Eng. 143 (6), 1–9. 10.1115/1.4050031/1097185 PMC8114905 33537729

[B91] Theodossiou S. K. Schiele N. R. (2019). Models of Tendon Development and Injury. BMC Biomed. Eng. 1 (32), 1–24. 10.1186/s42490-019-0029-5 32095779 PMC7039524

[B92] Thomopoulos S. Parks W. C. Rifkin D. B. Derwin K. A. (2015). Mechanisms of Tendon Injury and Repair. J. Orthop. Res. 33 (6), 832–839. 10.1002/JOR.22806 25641114 PMC4418182

[B93] Thornton G. M. Shrive N. G. Frank C. B. (2002). Ligament Creep Recruits Fibres at Low Stresses and Can Lead to Modulus-Reducing Fibre Damage at Higher Creep Stresses: A Study in Rabbit Medial Collateral Ligament Model. J. Orthop. Res. 20 (5), 967–974. 10.1016/S0736-0266(02)00028-1 12382961

[B94] Thorpe C. T. Chaudhry S. Lei I. I. Varone A. Riley G. P. Birch H. L. (2015). Tendon Overload Results in Alterations in Cell Shape and Increased Markers of Inflammation and Matrix Degradation. Scand. J. Med. Sci. Sports 25 (4), e381–e391. 10.1111/sms.12333 25639911

[B95] Thorpe C. T. Klemt C. Riley G. P. Birch H. L. Clegg P. D. Screen H. R. C. (2013). Helical Sub-structures in Energy-Storing Tendons Provide a Possible Mechanism for Efficient Energy Storage and Return. Acta Biomater. 9 (8), 7948–7956. 10.1016/J.ACTBIO.2013.05.004 23669621

[B96] Tohidnezhad M. Zander J. Slowik A. Kubo Y. Dursun G. Willenberg W. (2020). Impact of Uniaxial Stretching on Both Gliding and Traction Areas of Tendon Explants in a Novel Bioreactor. Ijms 21 (8), 2925–3019. 10.3390/ijms21082925 PMC7215532 32331279

[B97] Tom S. Parkinson J. Ilic M. Z. Cook J. Feller J. A. Handley C. J. (2009). Changes in the Composition of the Extracellular Matrix in Patellar Tendinopathy. Matrix Biol. 28 (4), 230–236. 10.1016/j.matbio.2009.04.001 19371780

[B98] Tse C. T. F. Keir P. J. (2020). External Compression and Partial Ischemia Decrease Human Finger Flexor Tendon and Subsynovial Connective Tissue Relative Motion. J. Orthop. Res. 38 (5), 1038–1044. 10.1002/JOR.24540 31793674

[B99] Tsuzaki M. Bynum D. Almekinders L. Yang X. Faber J. Banes A. J. (2003a). ATP Modulates Load-Inducible IL-1?, COX 2, and MMP-3 Gene Expression in Human Tendon Cells. J. Cell. Biochem. 89 (3), 556–562. 10.1002/JCB.10534 12761889

[B100] Tsuzaki M. Guyton G. Garrett W. Archambault J. M. Herzog W. Almekinders L. (2003b). IL-1β Induces COX2, MMP-1, -3 and -13, ADAMTS-4, IL-1β and IL-6 in Human Tendon Cells. J. Orthop. Res. 21 (2), 256–264. 10.1016/S0736-0266(02)00141-9 12568957

[B101] Vijven M. Wunderli S. L. Ito K. Snedeker J. G. Foolen J. (2021). Serum Deprivation Limits Loss and Promotes Recovery of Tenogenic Phenotype in Tendon Cell Culture Systems. J. Orthop. Res. 39 (7), 1561–1571. 10.1002/JOR.24761 32478872 PMC8359397

[B102] Wang H.-N. Huang Y.-C. Ni G.-X. (2020). Mechanotransduction of Stem Cells for Tendon Repair. Wjsc 12 (9), 952–965. 10.4252/WJSC.V12.I9.952 33033557 PMC7524696

[B103] Wang J. H.-C. (2006). Mechanobiology of Tendon. J. Biomechanics 39 (9), 1563–1582. 10.1016/J.JBIOMECH.2005.05.011 16000201

[B104] Wang J. H.-C. Guo Q. Li B. (2012). Tendon Biomechanics and Mechanobiology-A Minireview of Basic Concepts and Recent Advancements. J. Hand Ther. 25 (2), 133–141. 10.1016/J.JHT.2011.07.004 21925835 PMC3244520

[B105] Wang T. Chen P. Zheng M. Wang A. Lloyd D. Leys T. (2018). *In Vitro* loading Models for Tendon Mechanobiology. J. Orthop. Res. 36 (2), 566–575. 10.1002/jor.23752 28960468

[B106] Wang T. Lin Z. Day R. E. Gardiner B. Landao-Bassonga E. Rubenson J. (2013). Programmable Mechanical Stimulation Influences Tendon Homeostasis in a Bioreactor System. Biotechnol. Bioeng. 110 (5), 1495–1507. 10.1002/bit.24809 23242991

[B107] Wang T. Lin Z. Ni M. Thien C. Day R. E. Gardiner B. (2015). Cyclic Mechanical Stimulation Rescues Achilles Tendon from Degeneration in a Bioreactor System. J. Orthop. Res. 33 (12), 1888–1896. 10.1002/jor.22960 26123799

[B108] Wang T. Thien C. Wang C. Ni M. Gao J. Wang A. (2018). 3D Uniaxial Mechanical Stimulation Induces Tenogenic Differentiation of Tendon‐derived Stem Cells through a PI3K/AKT Signaling Pathway. FASEB J. 32 (9), 4804–4814. 10.1096/FJ.201701384R 29596022

[B109] Weinstein S. I. Yelin E. H. Watkins-Castillo S. I. (2014). Activity Limitation Due to Select Medical Conditions. Available at: https://www.boneandjointburden.org/fourth-edition/ic0/activity-limitation-due-select-medical-conditions (Accessed: June 30, 2021).

[B110] Wren T. A. L. Yerby S. A. Beaupré G. S. Carter D. R. (2001). Mechanical Properties of the Human Achilles Tendon. Clin. Biomech. 16 (3), 245–251. 10.1016/S0268-0033(00)00089-9 11240060

[B111] Wu Y. Han Y. Wong Y. S. Fuh J. Y. H. (2018). Fibre-based Scaffolding Techniques for Tendon Tissue Engineering. J. Tissue Eng. Regen. Med. 12, 1798–1821. 10.1002/term.2701 29757529

[B112] Xu H. Liu F. (2018). Downregulation of FOXP1 Correlates with Tendon Stem/progenitor Cells Aging. Biochem. Biophysical Res. Commun. 504 (1), 96–102. 10.1016/J.BBRC.2018.08.136 30170733

[B113] Zamboulis D. E. Thorpe C. T. Ashraf Kharaz Y. Birch H. L. Screen H. R. Clegg P. D. (2020). Postnatal Mechanical Loading Drives Adaptation of Tissues Primarily through Modulation of the Non-collagenous Matrix. eLife 9, 1–23. 10.7554/eLife.58075 PMC7593091 33063662

[B114] Zhang J. Nie D. Williamson K. McDowell A. Hogan M. V. Wang J. H.-C. (2020). Moderate and Intensive Mechanical Loading Differentially Modulate the Phenotype of Tendon Stem/progenitor Cells *In Vivo* . PLoS ONE 15 (12), e0242640–17. 10.1371/journal.pone.0242640 33373386 PMC7771689

[B115] Zhang J. Wang J. H.-C. (2009). Production of PGE2increases in Tendons Subjected to Repetitive Mechanical Loading and Induces Differentiation of Tendon Stem Cells into Non-tenocytes. J. Orthop. Res. 28 (2), a–n. 10.1002/JOR.20962 19688869

[B116] Zhang J. Wang J. H.-C. (2014). Prostaglandin E2 (PGE2) Exerts Biphasic Effects on Human Tendon Stem Cells. PLoS ONE 9 (2), e87706–12. 10.1371/JOURNAL.PONE.0087706 24504456 PMC3913640

[B117] Zhang K. Asai S. Yu B. Enomoto-Iwamoto M. (2015). IL-1β Irreversibly Inhibits Tenogenic Differentiation and Alters Metabolism in Injured Tendon-Derived Progenitor Cells *In Vitro* . Biochem. Biophysical Res. Commun. 463 (4), 667–672. 10.1016/J.BBRC.2015.05.122 PMC4496264 26051275

[B118] Zitnay J. L. Weiss J. A. (2018). Load Transfer, Damage, and Failure in Ligaments and Tendons. J. Orthop. Res. 36 (12), 3093–3104. 10.1002/JOR.24134 30175857 PMC6454883

