# Supplementary material for: Dynamic Load Model Systems of Tendon Inflammation and Mechanobiology
Source: Front Bioeng Biotechnol. 2022 Jul 15;10:896336. doi: 10.3389/fbioe.2022.896336 (PMC9335371; doi:10.3389/fbioe.2022.896336)
Supplement: Supplementary file 1 [file Table1.DOCX]

Supplementary Material

# Supplementary Figures


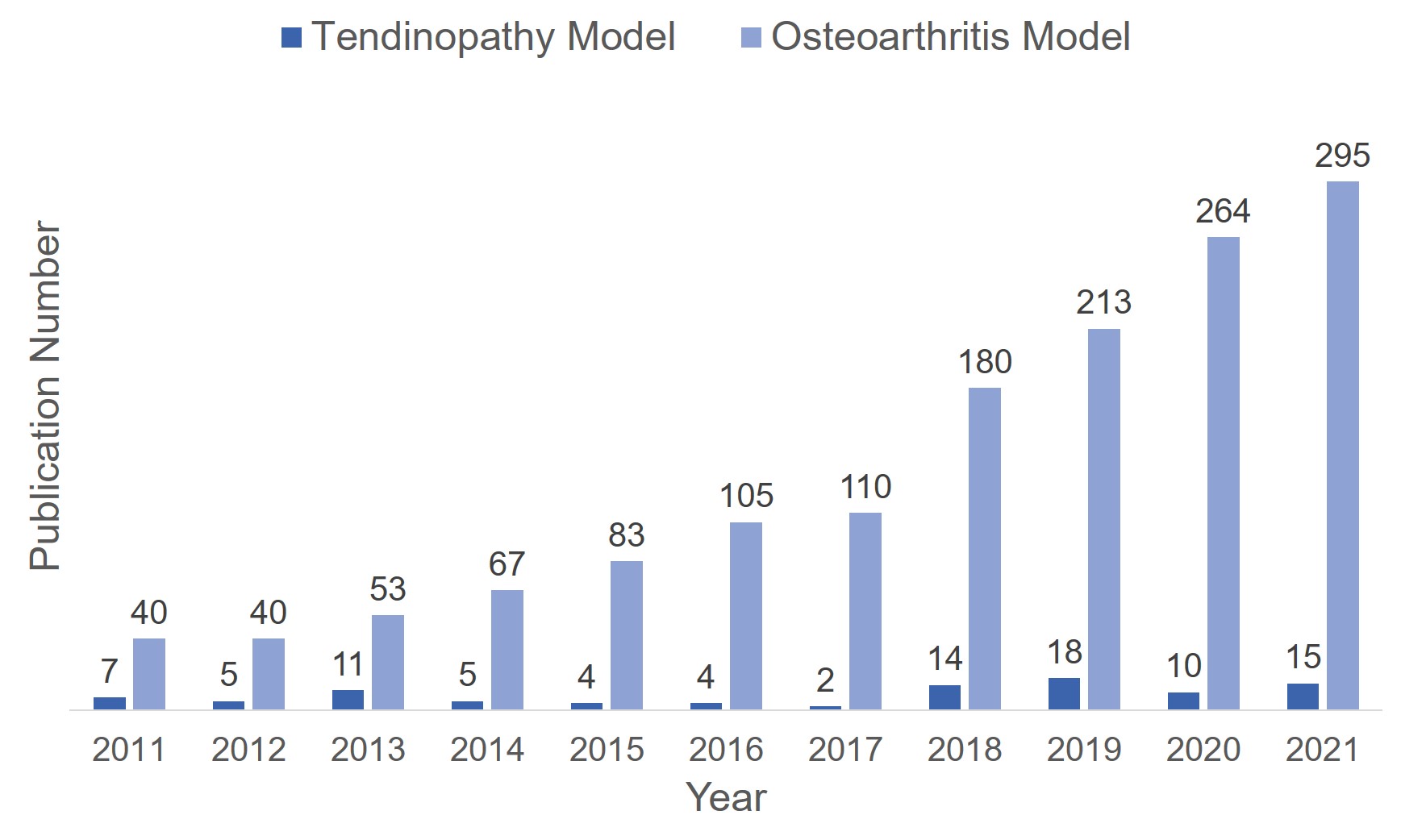


**Supplementary Figure 1**: Chart of publications by year from 2011-2021 based on key words and MeSH terms of “tendinopathy” AND “inflammation” AND “model” versus “osteoarthritis” AND “inflammation” AND “model” in PubMed databases.


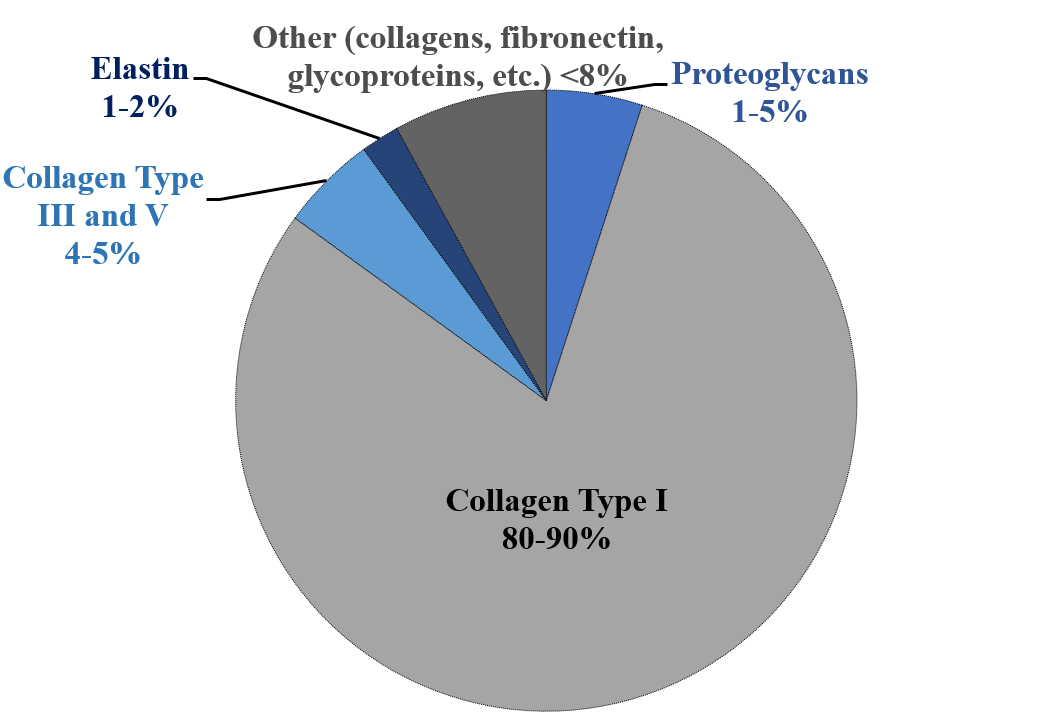


**Supplementary Figure 2**: Tendon extracellular matrix components with corresponding whole tendon dry weight contributions in percentages.
